# Performance of High-Throughput Sequencing for the Discovery of Genetic Variation Across the Complete Size Spectrum

**DOI:** 10.1534/g3.113.008797

**Published:** 2013-11-05

**Authors:** Andy Wing Chun Pang, Jeffrey R. MacDonald, Ryan K. C. Yuen, Vanessa M. Hayes, Stephen W. Scherer

**Affiliations:** * Department of Molecular Genetics, University of Toronto, Toronto, Ontario M5S 1A8, Canada; † The Centre for Applied Genomics, The Hospital for Sick Children, Toronto, Ontario M5G 0A4, Canada; ‡ J. Craig Venter Institute, San Diego, California 92121

**Keywords:** copy number variation, insertion/deletion, high-throughput sequencing, genome variation annotation

## Abstract

We observed that current high-throughput sequencing approaches only detected a fraction of the full size-spectrum of insertions, deletions, and copy number variants compared with a previously published, Sanger-sequenced human genome. The sensitivity for detection was the lowest in the 100- to 10,000-bp size range, and at DNA repeats, with copy number gains harder to delineate than losses. We discuss strategies for discovering the full spectrum of genetic variation necessary for disease association studies.

Insertion/deletion (indel, unbalanced change <100 bp) and copy number variation (CNV, unbalanced alteration 100 bp upwards) are increasingly observed to be important in development and disease (Lee and Scherer 2010; Weischenfeldt *et al.* 2013). However, in our experience, it has been difficult to detect indels and CNVs, even when the latest high-throughput sequencing (HTS) technologies are used (Pang *et al.* 2010). Although the detection of single-nucleotide variation by HTS seems sufficient (Lam *et al.* 2012b), the short reads of HTS limit the detection of larger and more complex genetic variants, and that limitation can hamper disease studies.

## Materials and Methods

To investigate the robustness of indel/CNV calling using HTS, we assessed data from commercial genome sequencing vendors and found that Complete Genomics (CG) (Drmanac *et al.* 2010) detected the greatest number of variants and yielded a more consistent and even variant size distribution (Supporting Information, Figure S1 and Table S1). To evaluate the quality of the CG variation (unbalanced genetic variants) profile, we chose to compare the structural variation data from a comprehensively characterized personal genome, namely the HuRef Standard (Levy *et al.* 2007; Pang *et al.* 2010), to 80 CG-sequenced genomes. One of the 80 genomes was HuRef, herein called HuRef CG (Table S2). The HuRef Standard assembly is of greater quality than HTS-generated genomes, since it was produced from high-accuracy Sanger-based sequencing of long mate-pair clone-end sequences. Using a combination of sequence- and microarray-based strategies, we detected 791,873 gains (insertions: size <100 bp or retrotransposons; duplications: size ≥100 bp) and losses (deletions) in HuRef relative to the National Center for Biotechnology Information reference assembly (Levy *et al.* 2007; Pang *et al.* 2010) (Table S3). Experimental validation confirmed 88% (184/210) of the variants (Levy *et al.* 2007; Pang *et al.* 2010). Details can be found in File S1.

## Results and Discussion

**Figure 1:**
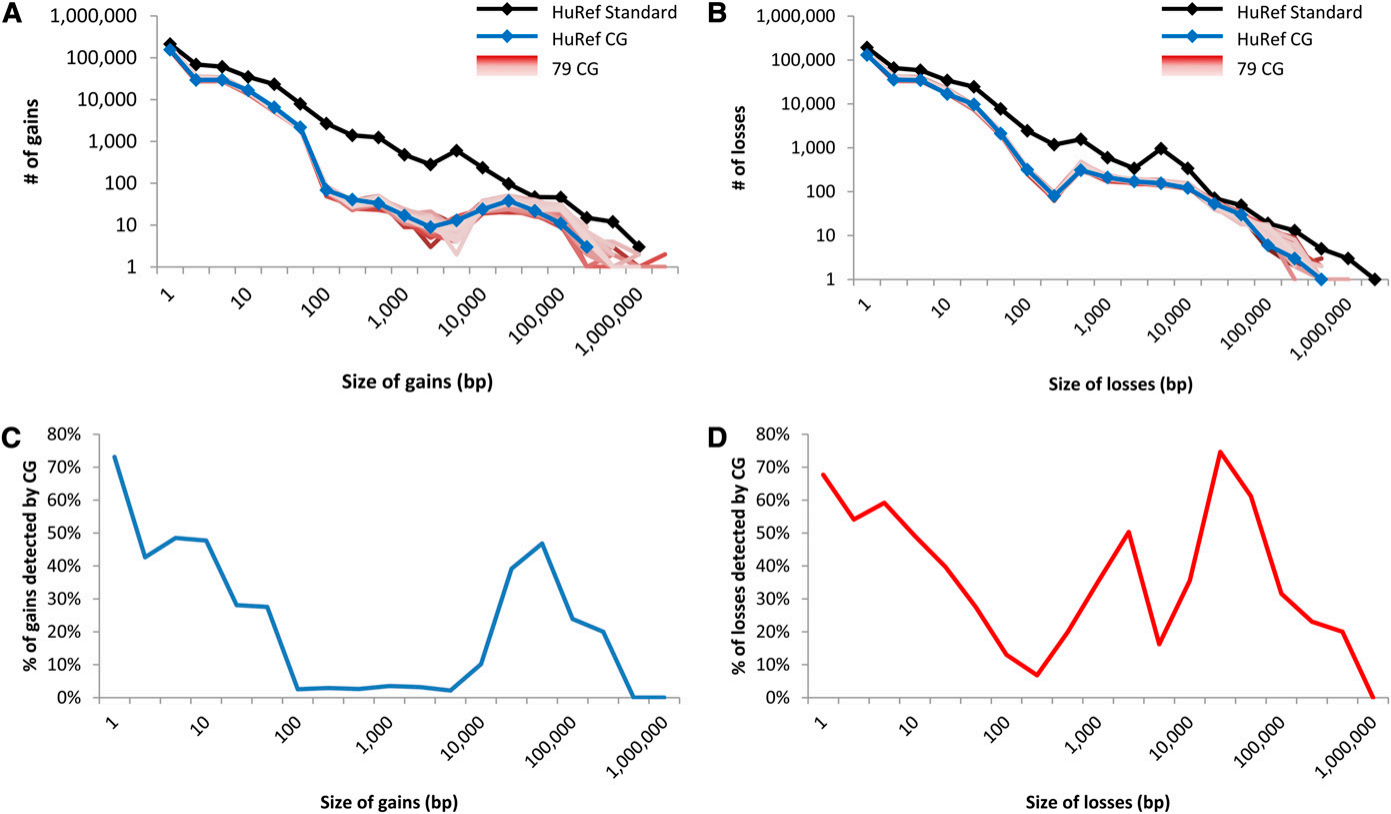
Variation distribution of genomes sequenced. The size distribution of nonredundant (A) gains and (B) losses detected in the HuRef and 79 other samples. The proportion of nonredundant (C) gains and (D) losses detected in HuRef by CG in comparison with HuRef Standard.

**Figure 2:**
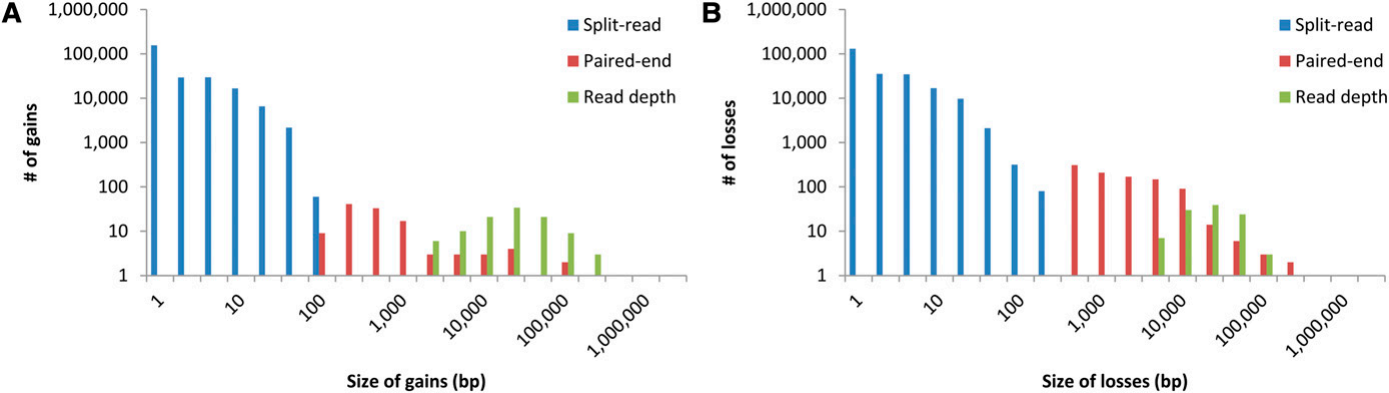
Size distribution of HuRef CG gains and losses detected by each discovery strategy examined: split-read, paired-end mapping and read depth. (A) Gains. (B) Losses.

First, by comparing the HuRef CG and HuRef Standard variation profiles, we noticed that short-read sequencing detected fewer calls and had substantial drops in discovery along the variation size spectrum (Figure 1, A and B). There were 241,033 gains and 230,737 losses in the HuRef CG data, which was a fraction of HuRef Standard’s 408,403 gains and 383,470 losses (Table S3). For losses, HuRef CG detected 60% of the total number of HuRef Standard losses whose size ranged from 1 to 100 bp, 30% of that from 100 to 10 kb, and 43% of that from >10 kb; for gains, HuRef CG detected 59% of that in HuRef Standard gains with the size ranged from 1 to 100 bp but only 7% of that from 100 to 10 kb and 21% of that from >10kb (Figure 1, C and D). CG used three primary approaches to detect gains and losses: split-read, paired-end and read depth (File S2 and Table S3). Unlike the uniform negative slope of the size distribution of variants annotated in the Sanger-based HuRef Standard (Figure 1, A and B), there were notable declines in sensitivity in the CG version, particularly for gains in the paired-end detection range, which spanned from 100 bp to 10 kb (Figure 2). As acknowledged by CG (Support & Community webpage), the paired-end detection approach had difficulty in calling variants at high-identity repeats, and calling novel insertion sequences relative to the National Center for Biotechnology Information reference.

To estimate false negatives in the CG profiles, we generated a compilation of variation from published studies (File S2, Figure S2, and Table S4). We identified a set of high confidence calls in the HuRef sample, by identifying HuRef Standard variants that were also detected in the population reference. We then examined the size distribution curves of HuRef CG variants against the curves representing the HuRef Standard variants also detected in the population reference, and we found that the HuRef CG curves were consistently below the curves of confirmed HuRef Standard. This analysis shows that there were variants missing in the HuRef CG profile; undercalling of gains greater than 100 bp was particularly severe (Figure S3). However, we emphasize that other short-read sequencing technologies also have similar problems, with large gains missing (Figure S1).

When comparing the HuRef CG data to the HuRef Standard, we determined that some of the missing gains and losses were from regions containing repeats. We found a notable reduction of calls in loci with retrotransposable repeats, tandem repeats and segmental duplications (two-tailed χ^2^ test; P < 2.2e-16) (Figure S4, A and B and C and D). It is difficult to align HTS reads to tandem repeat loci whose length can be longer than the short reads, and consequently, variant-detection at these loci is hampered. Similarly, short inserts can prevent aligning and assembling of paired reads to regions with retrotranposons and segmental duplications. These observations highlight the importance of having long reads and inserts for alignment and variant calling. As for centromeric and telomeric repeats, both Sanger sequencing and HTS have difficulty with these locations.

We evaluated false-positive results in the HuRef CG profile by comparing this data set to both the HuRef Standard and the profiles from the other 79 CG-sequenced genomes in this study, and we conservatively estimated that 11.4% of the HuRef CG gains and 3.9% of the losses could be false (File S2, Figure S5, and Table S5). Again, detection of gains was worse than losses.

From our comparison of the HuRef CG and HuRef Standard datasets, we observed that CG also had notable strengths. First, the HuRef CG loss size distribution was fairly uniform when compared to the expected HuRef Standard (Figure 1B). Second, CG was highly precise in determining variant size, with the exception of overcalling by the read-depth approach (Figure S6). Increasing the sequence coverage plus decreasing the bin-size may reduce this overestimation. Finally, the HuRef CG variant profiles were similar to the profiles of the other 79 CG genomes, highlighting consistency across experiments (File S2 and Figure 1, A and B).

Taking advantage of the availability of a comprehensive set of variation from a fully sequenced genome, we have analyzed the performance of detecting insertion and deletion by a HTS technology. Overall, we conclude that only a fraction of kown variation was captured, with notable shortcomings in detecting insertions and duplications in the 100-bp to 10-kb size range, and at repetitive DNA sequences. Many of these deficiencies are associated with short reads and insert lengths (File S2, Figure 1, A and B, Figure S4 and Figure S7, Table S6 and Table S7). Generating longer reads (Loomis *et al.* 2013) or libraries of multiple insert lengths can mitigate these shortcomings. Greater depth of coverage can also partially recover some of the missing calls. Among our 80 CG-sequenced samples (File S2, Figure S8 and Figure S9), we noticed that the sequenced-depth and the number of variants reported were positively correlated (gains: R = 0.36, *P* = 0.00097; losses: R = 0.41, *P* = 0.00017; Figure S10). Computationally, one should continue to apply multiple complementary variant detection strategies: split-read, paired-end, read depth, and one-end-anchor approaches (Hajirasouliha *et al.* 2010). Moreover, whole-genome assembly comparison approach should be considered (Khaja *et al.* 2006; Levy *et al.* 2007), as our analysis has shown that this approach can yield the greatest number, type and size range of variation (Table S3). However, current *de novo* assembly of short sequences is often restricted by the presence of repeats. A possible solution is a hybrid assembly constructed with a mixture of shallow coverage (~5×) of mate-pair long-reads with deeper coverage (~25×) of paired-end short-reads (Schatz *et al.* 2010; Gnerre *et al.* 2011). Alternatively, sequencing can be performed in conjunction with microarray or single-molecule physical mapping (Lam *et al.* 2012a) to detect larger variation. Physical mapping or other complexity-reduction processes [*e.g.*, Long Fragment Read (Peters *et al.* 2012)] should improve alignment and the accuracy of variant discovery. Finally, some common variants (minor allele frequency >5%) that are missed by HTS could be imputed by nearby tag SNPs, although it may not be applicable to some rare variants as it has been shown that ~20% of biallelic CNVs cannot be readily captured (Mills *et al.* 2011). Ultimately, if HTS is to become a primary technology in clinical laboratories it will further benefit from improvement, particularly in capturing rare indels, CNVs and more complex rearrangements that are associated with diseases.

## Supplementary Material

Supporting Information
