# Supplementary material for: Performance of High-Throughput Sequencing for the Discovery of Genetic Variation Across the Complete Size Spectrum
Source: G3 (Bethesda). 2013 Nov 5;4(1):63–5. doi: 10.1534/g3.113.008797 (PMC3887540; doi:10.1534/g3.113.008797)
Supplement: Supporting Information [file supp_4_1_63__index.html]

Performance of High-Throughput Sequencing for the Discovery of Genetic Variation Across the Complete Size Spectrum — Supporting Information 

# Performance of High-Throughput Sequencing for the Discovery of Genetic Variation Across the Complete Size Spectrum

## Supporting Information for Pang *et al.*, 2014

**Files in this Data Supplement:**

- Supporting Information - Figures S1-S10, Files S1-S2, and Tables S1-S7 (PDF, 1 MB)
- Figure S1 - The size distributions of reported DNA gains and losses in published personal genome sequencing studies. (PDF, 350 KB)
- Figure S2 - Size distribution of gains and losses identified in 18 studies that constitute the population reference data set. (PDF, 327 KB)
- Figure S3 - The size distribution of HuRef CG variation and HuRef Standard variation that was confirmed by published studies. (PDF, 343 KB)
- Figure S4 - Proportion of HuRef Standard and HuRef CG gains and losses residing in repetitive regions. (PDF, 345 KB)
- Figure S5 - Overall concordance statistics between HuRef Standard and HuRef CG variation sets. (PDF, 343 KB)
- Figure S6 - Complete Genomics variant breakpoint estimation. (PDF, 333 KB)
- Figure S7 - The percentage of HuRef CG gains and losses between 100 bp and 100 kb residing in retrotransposable repeats, tandem repeats, segmental duplications, centromeric and telomeric repeats. (PDF, 312 KB)
- Figure S8 - Density distribution of non-redundant Complete Genomics variants found in our 79-sample cohort along chromosomal locations. (PDF, 874 KB)
- Figure S9 - Frequency of gains and losses detected in the 79 Complete Genomics cohort. (PDF, 314 KB)
- Figure S10 - Positive correlation between the depth of coverage and the number of gains and losses detected among the 80 samples sequenced by CG. (PDF, 315 KB)
- File S1 - Supplementary Materials and Methods (PDF, 321 KB)
- File S2 - Supplementary Results (PDF, 330 KB)
- Table S1 - Summary of variation results in several personal genomes (.xlsx, 10 KB)
- Table S2 - Summary information of genomes sequenced in the current study (.xlsx, 12 KB)
- Table S3 - Gains and losses detected in the HuRef genome by different methods (.xlsx, 11 KB)
- Table S4 - Summary of variation results from published population studies (.xlsx, 10 KB)
- Table S5 - The proportion of HuRef CG variants that were also detected in our 79 Complete Genomics-sequenced samples (.xlsx, 8 KB)
- Table S6 - Comparison of the HuRef Standard-only variants with records annotated as mobile element insertions by Complete Genomics (.xlsx, 9 KB)
- Table S7 - Comparison of the HuRef Standard-only variants with records annotated as hypervariable and invariant by Complete Genomics (.xlsx, 10 KB)
