# Supplementary material for: Performance of High-Throughput Sequencing for the Discovery of Genetic Variation Across the Complete Size Spectrum
Source: G3 (Bethesda). 2013 Nov 5;4(1):63–5. doi: 10.1534/g3.113.008797 (PMC3887540; doi:10.1534/g3.113.008797)
Supplement: Supporting Information [file supp_g3.113.008797_008797SI.pdf]

G3: Genes | Genomes | Genetics

Performance of high-throughput sequencing for the discovery of genetic variation across the complete size spectrum

Andy Wing Chun Pang[1,2], Jeffrey R. MacDonald[2], Ryan K. C. Yuen[2], Vanessa M. Hayes[3], Stephen W. Scherer[1,2,*]

[1] Department of Molecular Genetics, University of Toronto, Toronto, Ontario M5S 1A8, Canada
[2] The Centre for Applied Genomics, The Hospital for Sick Children, Toronto, Ontario M5G 0A4, Canada
[3] J. Craig Venter Institute, 10355 Science Centre Drive, San Diego, California 92121, USA
[*] Corresponding author

Email addresses:
AWCP: andypang@sickkids.ca
JRM: jmacdonald@sickkids.ca
RKCY: ryan.yuen@sickkids.ca
VMH: vhayes@jcvi.org
SWS: stephen.scherer@sickkids.ca

DOI: 10.1534/g3.113.008797

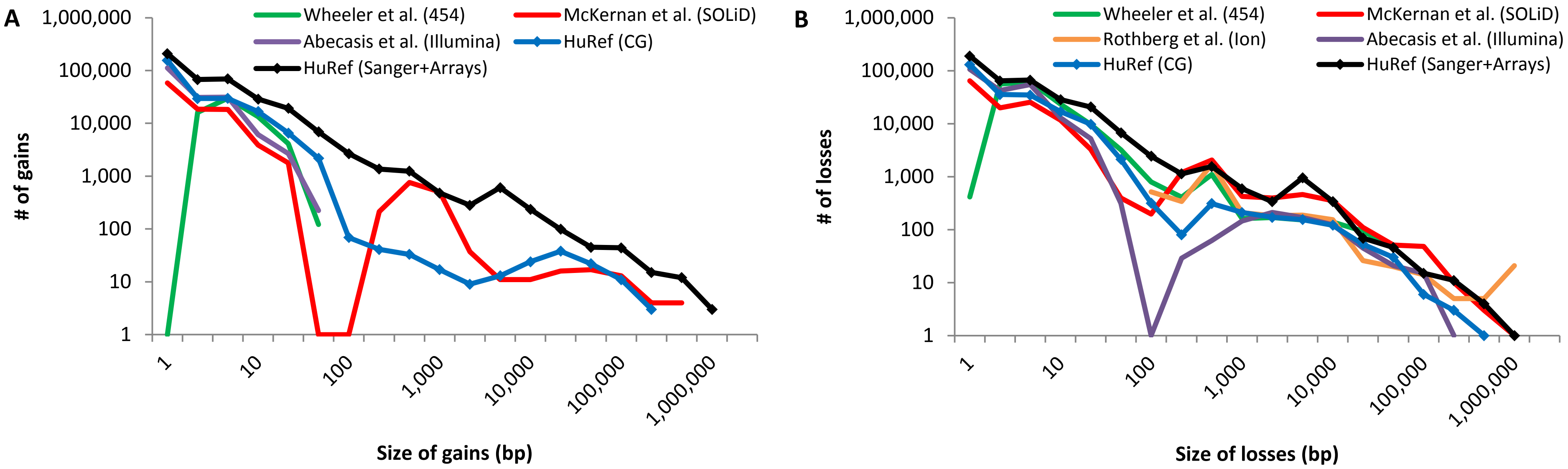

Figure S1 The size distributions of reported DNA gains and losses in published personal genome sequencing studies. These diagrams show the relative uniformity of CG variants across the size spectrum. In Wheeler et al., insertions identified by intra-read alignment would be limited by the size of the 454 sequencing reads; hence, large insertions beyond the read length were not detected (WHEELER *et al.* 2008). McKernan et al. used SOLiD sequencing and microarrays to detect variation in a Yoruba individual NA18507 (MCKERNAN *et al.* 2009). They detected small variants based on split-reads and large variants based on mate-pair mapping and microarrays, but failed to find medium size gains. Rothberg et al. performed whole genome sequencing using the Ion Torrent technology, but only reported deletions of at least 50 bp in size (ROTHBERG *et al.* 2011). Primarily sequenced by Illumina, Abecasis and colleagues detected variation in the sample NA18507 using a multitude of calling algorithms (ABECASIS *et al.* 2012). However, for large variation, only deletions were reported. The number of calls is also listed in Table S1. From these figures, CG yielded the most consistent calling pattern across the size spectrum among HTS genomes, thus justifying our selection of CG for comparison in this current study.

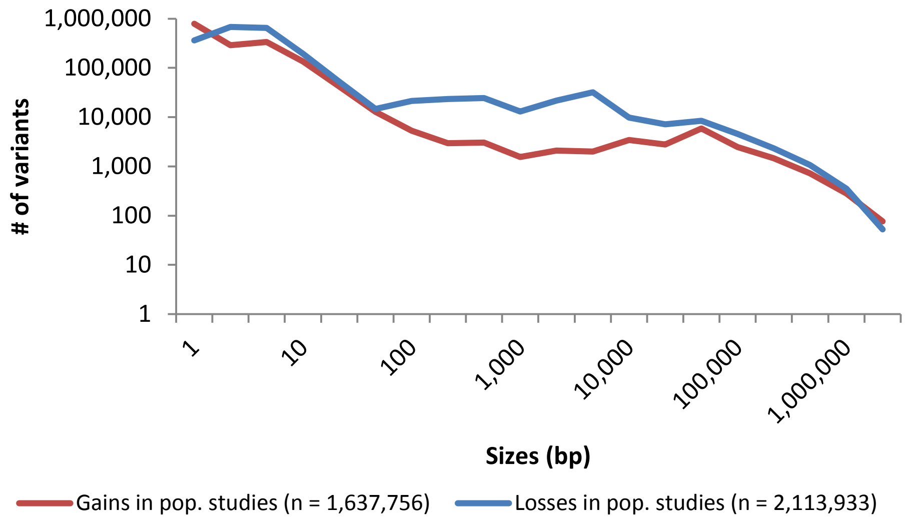

Figure S2 Size distribution of gains and losses identified in 18 studies that constitute the population reference data set. A summary of the data is also listed in Table S4.

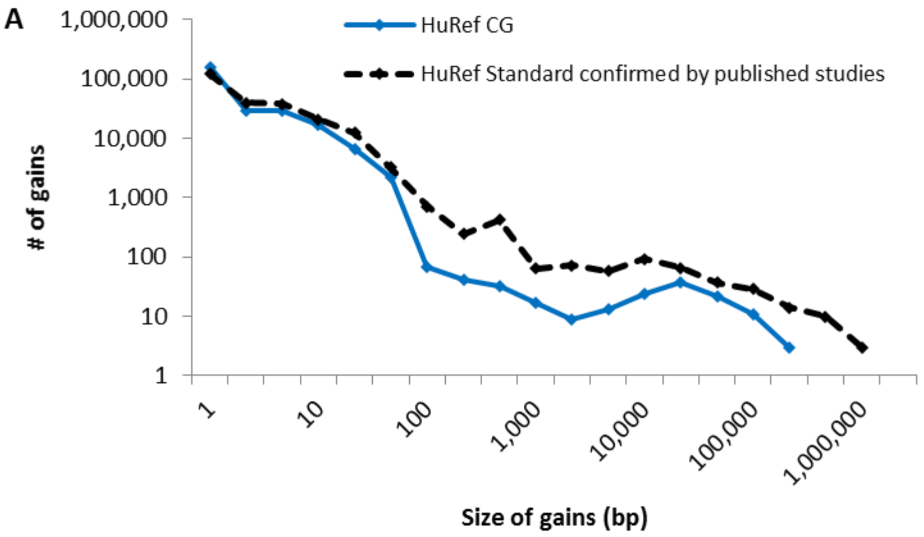

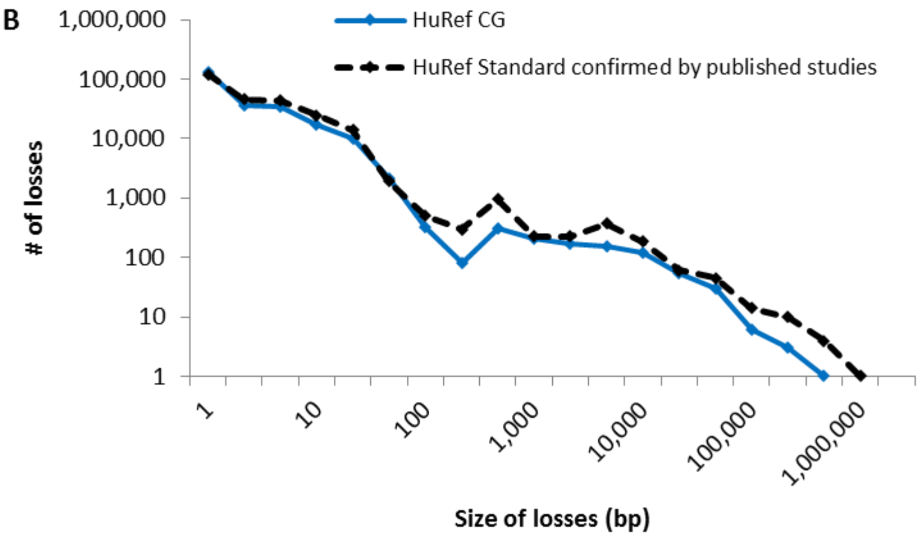

Figure S3 The size distribution of HuRef CG variation and HuRef Standard variation that was confirmed by published studies. The distributions for gains and losses are shown in plots (A) and (B), respectively. Note the resemblance of both plots to Figure 1 A and B, and that the confirmed HuRef Standard curves were consistently equal to or above the HuRef CG curves.

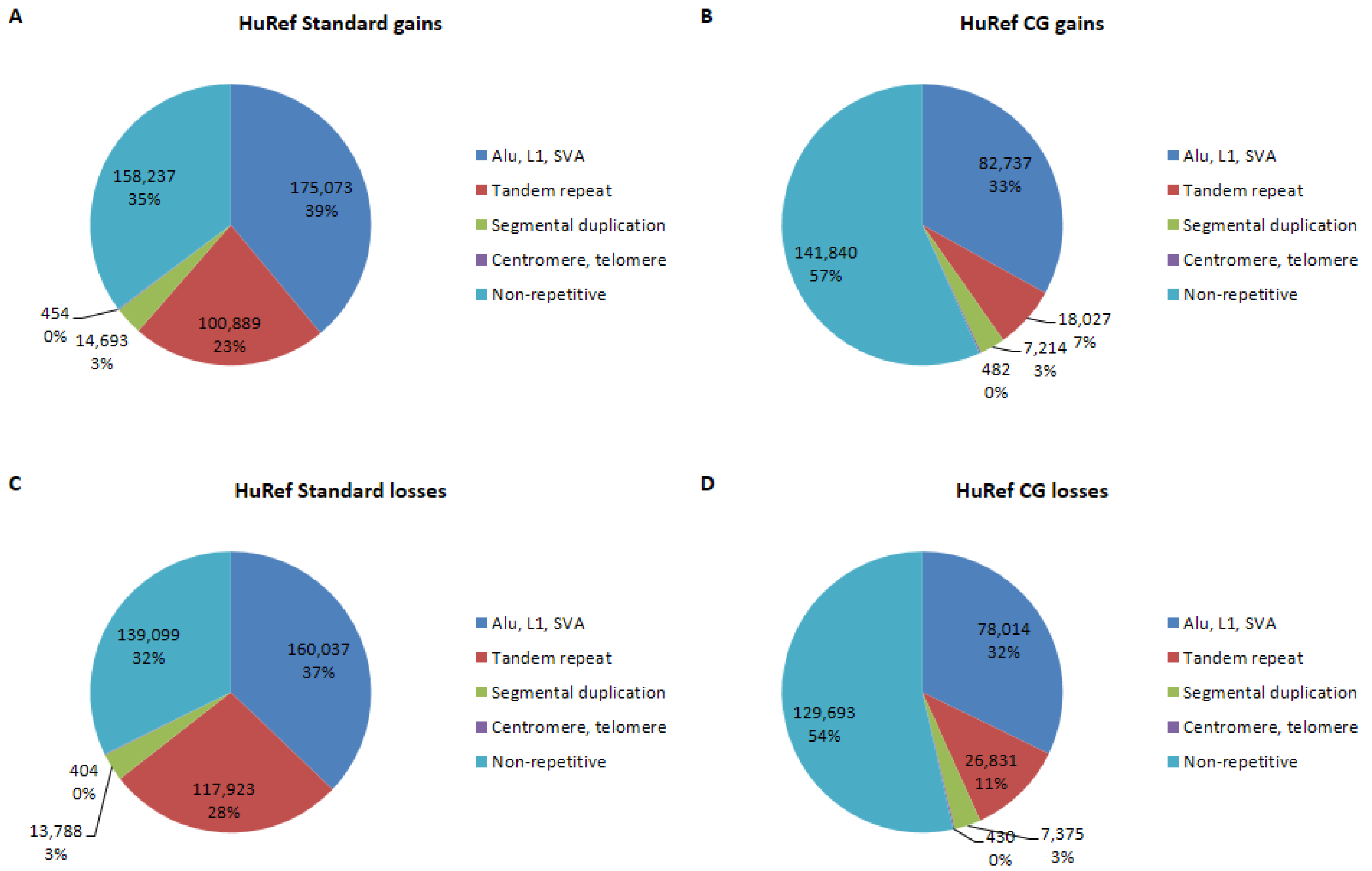

Figure S4 Proportion of HuRef Standard and HuRef CG gains and losses residing in repetitive regions. (A) The proportion of HuRef Standard gains residing in retrotransposable repeats, tandem repeats, segmental duplications, centromeric and telomeric repeats, and non-repetitive regions. (B) HuRef CG gains and proportion of repeats. (C) HuRef Standard losses. (D) HuRef CG losses. Note the under-representation of HuRef CG calls in retrotransposons, tandem repeats and segmental duplications when compared to the HuRef Standard profile. Finally, the sum of the numbers reported in these plots exceeds the total number of variants, because it is possible that one variant overlap with more than two types of repeats.

HuRef CG total	
# gains	241,033
# losses	230,737

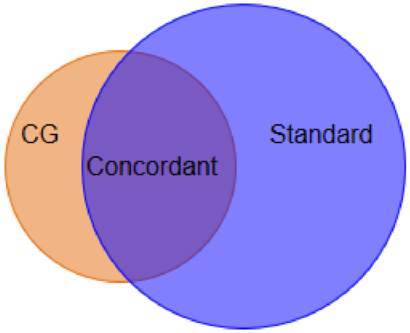

HuRef Standard total	
# gains	408,403
# losses	383,470

HuRef CG-specific*	
# gains (%)	98,665 (40.93)
# losses (%)	70,345 (30.49)

Concordant*	
# gains (%)	142,368 (59.07)
# losses (%)	160,392 (69.51)

HuRef Standard-specific**	
# gains (%)	265,858 (65.10)
# losses (%)	222,549 (58.04)

* Percentage is with respect to HuRef CG total

** Percentage is with respect to HuRef Standard total

Figure S5 Overall concordance statistics between HuRef Standard and HuRef CG variation sets.

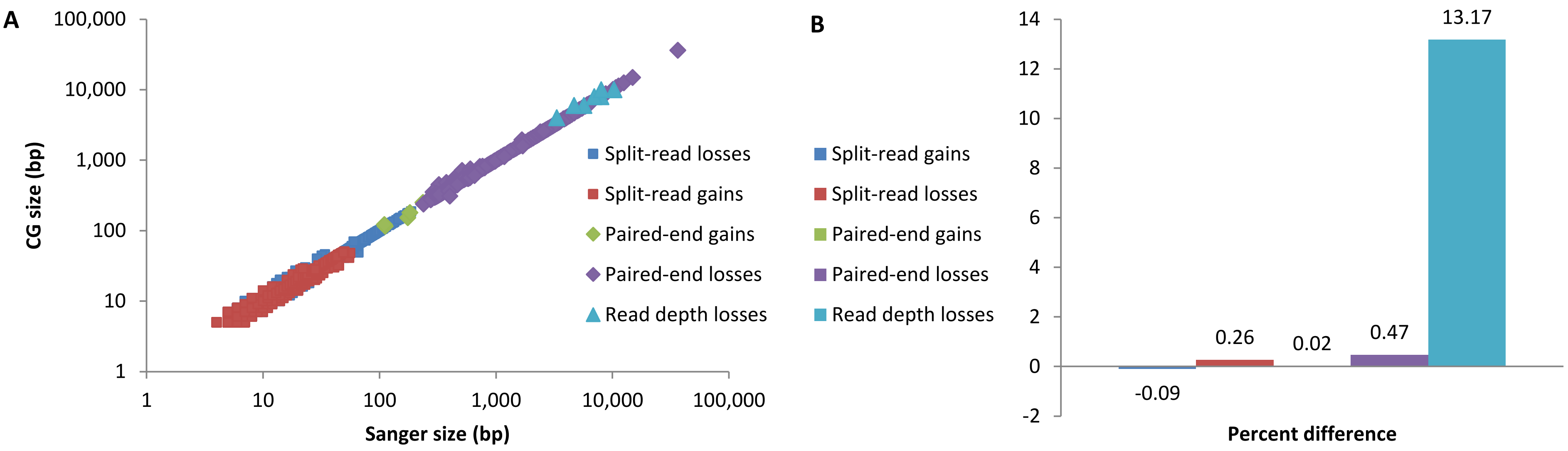

Figure S6 Complete Genomics variant breakpoint estimation. (A) shows the tight size correlation between HuRef CG variants (> 5bp) and the corresponding breakpoint-refined HuRef Standard variants, and (B) displays the average percentage of size difference between HuRef CG and HuRef Standard.

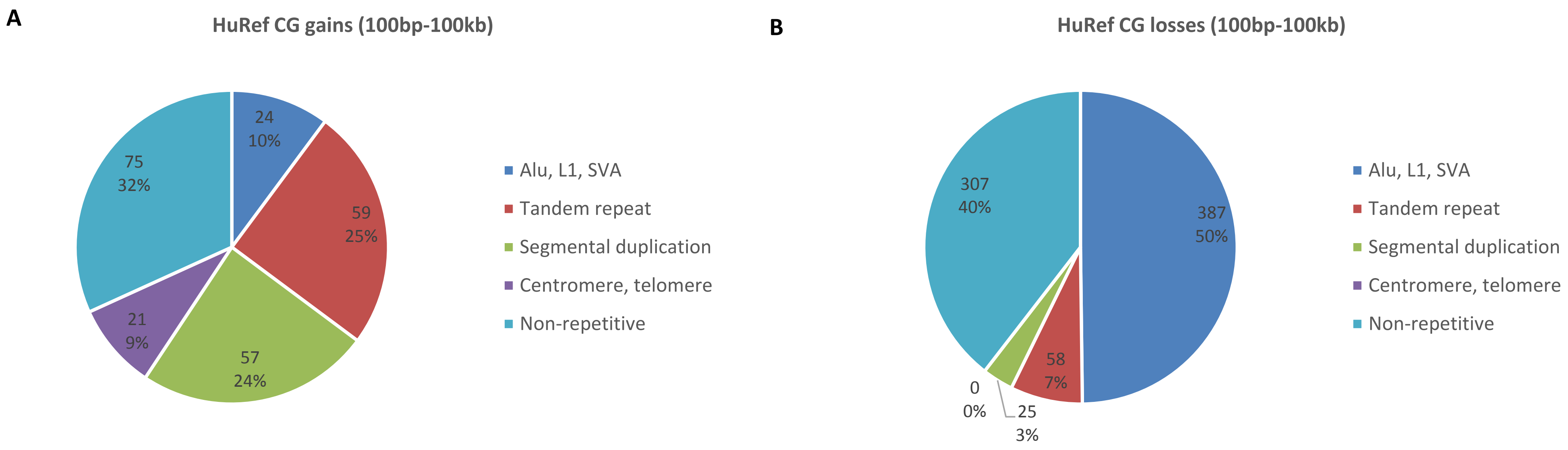

Figure S7 The percentage of HuRef CG gains and losses between 100 bp and 100 kb residing in retrotransposable repeats, tandem repeats, segmental duplications, centromeric and telomeric repeats. The depletion of insertions reported in retrotransposable elements has been known and this situation has also been acknowledged by Complete Genomics. Surprisingly, there were significant elevations in the number of HuRef CG gains versus losses residing in other types of repeats (tandem repeat and segmental duplication). However, these repeats could also be problematic for short-read alignments. The enrichment of gains over losses found in these loci could explain why a lower CG paired-end and CG read depth confirmation rate was observed for gains compared to losses. (A) HuRef CG gains. (B) HuRef CG losses.

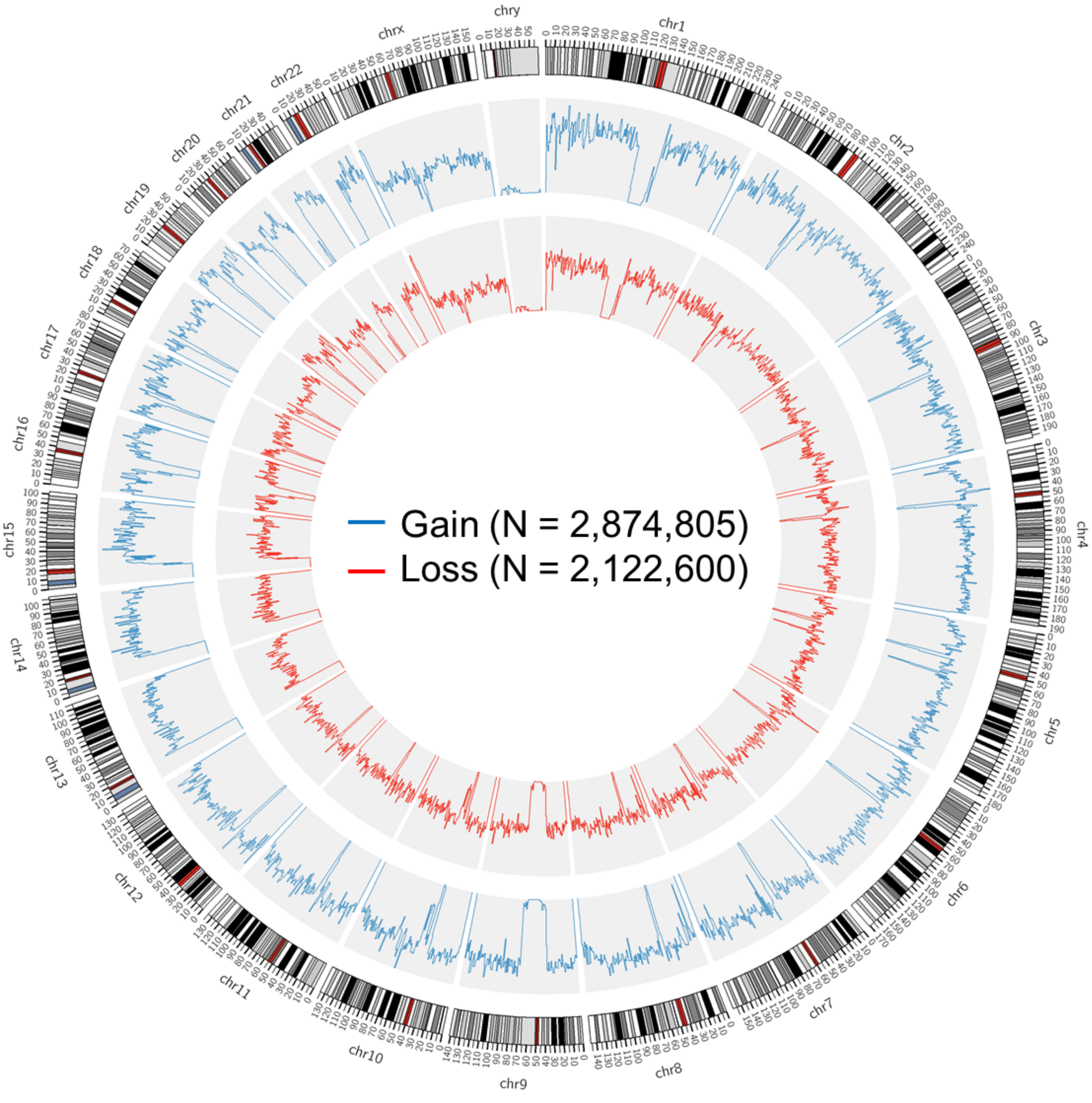

Figure S8 Density distribution of non-redundant Complete Genomics variants found in our 79-sample cohort along chromosomal locations. The density of gains is presented by the blue track, while losses by the red (number of non-redundant variant per megabase). This provides a snapshot of the distribution of calls along chromosomes. For example, the overall profiles between gain and loss are similar, and the density at repetitive loci (centromeres, telomeres, p-arm of acrocentric chromosomes, chromosome Y) and are notably low. Centromeres are shown in red in the ideogram.

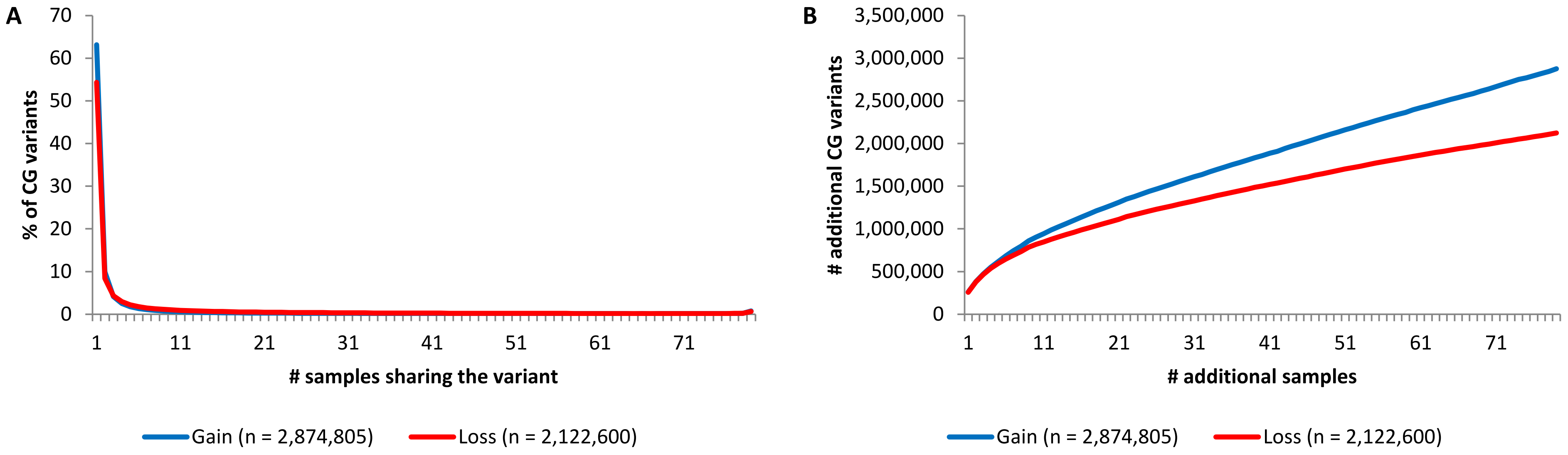

Figure S9 Frequency of gains and losses detected in the 79 Complete Genomics cohort. (A) The frequency of gains and losses found in the 79 samples sequenced by Complete Genomics. (B) Cumulative frequency distribution showing the number new variants obtained with the sequencing of each additional DNA sample.

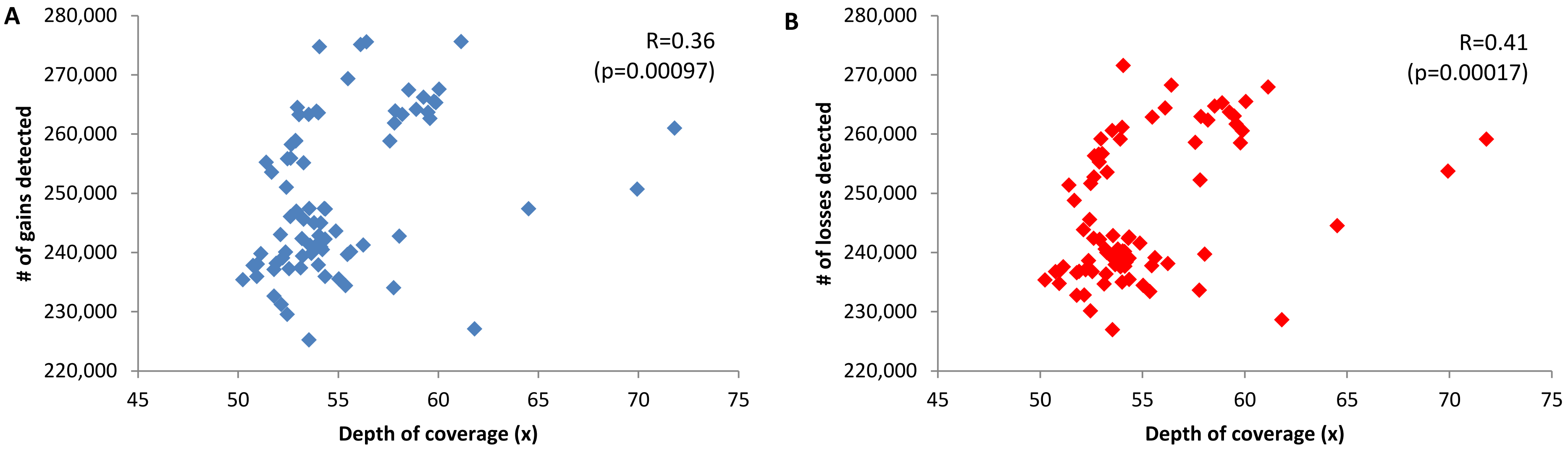

Figure S10 Positive correlation between the depth of coverage and the number of gains and losses detected among the 80 samples sequenced by CG. (A) Gains. (B) Losses.

File S1

Supplementary Materials and Methods

Complete Genomics sequencing experiment and data analysis

As required by Complete Genomics (CG) (Drmanac *et al.* 2010), 10 ug of non-degraded DNA was provided for sequencing. The sequencing experiments and variation-detection of the genomes of Craig Venter (HuRef) (Levy *et al.* 2007) and 79 clinically unaffected Caucasians were performed in house at CG. Paired-end reads were aligned to the Genome Reference Consortium human genome reference GRCh37. A summary of the sequencing experiments is shown in Supplementary Table 2.

The variant calls were extracted from CG files using custom Perl scripts. The variation set used for this study was obtained from three primary sources: (i) The CG split-read insertion and deletion records were extracted from the masterVarBeta file. (ii) The paired-end set was obtained from the SV/highConfidenceSVEventsBeta file, and only deletion, distal and tandem duplications were extracted. (iii) The read depth data was taken from the CNV/cnvSegmentsDiploidBeta file. The records of hypervariable or invariant were not included in our final data set for subsequent analysis, because the assignment of calls as gain or loss was not provided. However, we did perform additional analysis incorporating these calls in the HuRef CG (the HuRef CG) dataset to try to improve the concordant rate with the HuRef Sanger + Array standard (the HuRef Standard) (Levy *et al.* 2007; Pang *et al.* 2010). See Supplementary Results *Analysis of MEI, hypervariable and invariant CG records* section for more information.

CG also explicitly searched for potential mobile element insertions (MEIs), and they were listed in MEI/mobileElementInsertionsBeta file. We did not include these calls in the final variant set, as the variant size was not annotated. Although the file did annotate the start and end of insertion fragment within the consensus sequence of the mobile element, the information might not necessarily represent the size of the complete insertion sequence. There could be sequences within an insertion fragment that could not be aligned to the mobile element consensus sequence. So, entries in the file were not included in the CG final variant set. Nonetheless, we did compare a subset of these MEI calls in the HuRef sample with the calls in the HuRef Standard, and the results are listed in Supplementary Results *Analysis of MEI, hypervariable and invariant CG records* section.

Non-redundant Complete Genomics variant set generation

To generate a non-redundant set of variation, we combined the split-read, paired-end and read depth records. First we searched for overlap between the paired-end and read depth sets requiring that the variants to be the same type (i.e. duplication or deletion) and that they shared a minimum of 50 % reciprocal size overlap. Next, we used the same criteria to merge this dataset with the split-read calls. For those calls that were determined to be the same variant, we recorded the one with a better size/boundary estimate, with preference given to split-read, then paired-end, then read depth.

Population reference data set

We compiled a non-redundant set of calls from 18 published studies (Jakobsson *et al.* 2008; Kidd *et al.* 2008; McCarroll *et al.* 2008; Perry *et al.* 2008; Wheeler *et al.* 2008; Alkan *et al.* 2009; Altshuler *et al.* 2010; Conrad *et al.* 2010; Durbin *et al.* 2010; Itsara *et al.* 2010; Ju *et al.* 2010; Kidd *et al.* 2010a; Kidd *et al.* 2010b; Teague *et al.* 2010; Tong *et al.* 2010; Mills *et al.* 2011; Pinto *et al.* 2011; Abecasis *et al.* 2012) for comparison with the HuRef data. A summary of these studies can be found in Supplementary Table 4. This list consisted of studies that used a variety of variation-detection methodologies, ranging from NGS sequencing, Sanger sequencing and Sanger fosmid mate-pair mapping, high density SNP genotyping microarrays, high resolution comparative genome hybridization microarrays, and optical mapping. To determine if two calls correspond to the same underlying event, we used a strict 70 % reciprocal size overlap criterion.

Genomic features data set

The positions of retrotransposable, centromeric and telomeric repeats were taken from Repeat Masker (Smit 1996-2010). Segmental duplications information was obtained from the University of California, Santa Cruz (UCSC) database. Tandem repeats annotation was taken from Tandem Repeats Finder (Benson 1999).

File S2

Supplementary Results

Examination of HuRef variants called by different Complete Genomics detection approaches

We investigated the confirmation rate among the detection approaches of CG. It used three primary approaches to detect non-SNP variants: paired-end, split-read and read depth. The first approach used by CG is the paired-end mapping method. When paired sequences from an insert library of defined sizes created from genomic DNA from a test individual are sequenced, they are then aligned to a reference assembly. The distance between the pair is then compared with the expected size of the insert. Any discrepancy in distance between the observed and expected size of the insert would indicate a putative insertion or deletion (KORBEL *et al.* 2007; KIDD *et al.* 2008; HORMOZDIARI *et al.* 2009). The split-read approach will detect insertions and deletions by identifying intra-alignment gaps (MILLS *et al.* 2006; YE *et al.* 2009). Finally, the read depth approach is used to identify duplications where there is a significantly elevated number of alignments compared to diploid regions, while deleted regions are identified when there is a significantly reduced number of mapped reads (CHIANG *et al.* 2009; ABYZOV *et al.* 2011).

Among the three detection approaches employed by CG, the split-read approach was used to call small variants, paired-end approach for medium to large variants, and read depth approach for large ones (Fig. 2, Supplementary Table 3). In the HuRef CG dataset, there was no overlap between variants called by the split-read and the other approaches, mainly because the size of split-read calls was below others' detection limits. As for the larger variation, the concordant rate between paired-end and read depth sets was modest; their consensus was two for the gains and 45 for the losses. Interestingly, there was an elevated level of overlap with segmental duplications and tandem repeats in the gains compared to the losses in the 100 bp to 100 kb size range (Figure S7). The difficulty to localize variants by short reads mapping to repeats could explain the lower cross-detection-approach confirmation rate of the gains.

Estimation of Complete Genomics variant-detection sensitivity

We compared the HuRef Standard and HuRef CG variants with published variation studies, which constituted our population reference. These studies have used multiple variant-detection methods: high-throughput sequencing (HTS), Sanger read-trace, Sanger fosmid-end mapping, microarrays and optical mapping (Supplementary Table 4), and from these, we compiled 1,637,756 non-redundant gains and 2,113,933 non-redundant losses, whose size distributions are displayed in Supplementary Figure 2. The uneven size distributions of the population reference suggested a detection bias to variants of certain sizes. Even though we incorporated studies using a wide range of genomic approaches, there were still shortcomings at certain size ranges, especially between 100 bp to 10 kb. Despite the notable shortcomings in the population reference, we compared it with the HuRef Standard and HuRef CG variation profiles.

First, the size distribution curves representing those HuRef Standard variants also detected in the population reference were consistently at or above the overall HuRef CG curves, across the entire size spectrum (Supplementary Fig. 3), indicating that there were variants missing in the HuRef CG profile.

Second, we examined the proportion of HuRef CG-only and HuRef Standard-only variants confirmed by the population reference. We found that a similar proportion of HuRef CG-only (44.9 % gains and 61.3 % losses) and HuRef Standard-only variants (40.6 % gains and 50.0 % losses) were in accord with the population reference. So, both data sets contained genuine variants that were undetectable by the other. We want to emphasize that our usage of population reference was for estimating sensitivity, not false discovery. Any discordance could be due to sample difference or methodological difference in variation detection. Furthermore, the population reference was incomplete, with a notable plateau at the 100 bp to 10 kb range (Supplementary Fig. 2), and this in turn could lower the concordant rate in both HuRef variants sets.

Estimation of Complete Genomics variant-detection specificity and false discovery rate

While there was a good overall concordance (64.2%) for the HuRef CG calls with the HuRef Standard, the specificity to detect gains was lower than that for losses. We found that 59.1% (142,368/241,033) of gains and 69.5% (160,392/230,737) of losses called by HuRef CG were concordant (70% reciprocal size overlap) with the HuRef Standard (Supplementary Fig. 5).

Since there could be variants discoverable only by the CG technology, we refined the false discovery rate by further comparing the HuRef CG profile with those from the other 79 CG-sequenced genomes in this study. By comparing with samples that were also sequenced on the same platform, we removed the confounding issue of methodological difference in variation detection. Among the HuRef CG-only variants not detectable by HuRef Standard, there were *(I)* 28,020 gains and 9,904 losses that were also not seen in the other 79 samples (Supplementary Table 5). These calls could be false discoveries in the HuRef CG experiment or rare/private HuRef CG variants undetectable by the HuRef Standard.

On the other hand, among the set of HuRef CG variants that were concordant with the HuRef Standard, only *(II)* 937 (of 142,368; or 0.66%) gains and 1,996 (of 160,392; or 1.24%) losses were also not supported by the 79 individuals (Supplementary Table 5), and we reasoned that these proportions of calls should represent high confidence rare/private HuRef variants.

We then applied percentage from *(II)* to the calls in HuRef CG-only (not confirmed in HuRef Standard) and estimated the expected number of these calls to be private.

98,665 gains * 0.66% = 651 expected number of rare/private gains
70,345 losses * 1.24% = 872 expected number of rare/private losses*…(III)*

Therefore by taking the difference between *(I)* and *(III)*, we estimated that 27,369 or 11.35% (= [28,020 – 651] / 241,033 * 100) HuRef CG gains, and 9,032 or 3.91% (= [9,904 – 872] / 230,737 * 100) HuRef CG losses could be false discoveries. Once again, similar to Supplementary Figure 5, we observed that the specificity to detect gains was lower than losses.

However, we emphasize that these are only estimates, as there are other types of false calls unaccounted for. One type is false variation repeatedly called in all CG experiments. One such example was the 16.8 Mb HuRef deletion artifact found by paired-end mapping method of CG, which was also repeatedly called in all 79 genomes in our sample cohort (Supplementary Table 3). This large-size call was likely false as it had not been confirmed by the HuRef Standard or cytogenetic experiments (LEVY *et al.* 2007).

Finally, we examined additional HuRef CG records for concordance (mobile element insertions, hypervariable and invariant calls), but the improvement was insignificant. See the section *Analysis of MEI, hypervariable and invariant Complete Genomics records* in Supplementary Results.

Analysis of MEI, hypervariable and invariant Complete Genomics records

From our concordance analysis of comparing the HuRef CG data with the HuRef Standard data, we determined that 142,368 (59.1 %) gains and 160,392 (69.5 %) losses called by CG were also concordant with the HuRef Standard. In other words, there were 265,858 gains and 222,549 losses specific to the HuRef Standard and undetectable by CG. We then included additional potentially relevant but lower confidence CG calls. For the following exercises, we examined the number CG calls that might correspond to calls that were HuRef Standard-only.

First, we examined the CG MEI variants in the file MEI/mobileElementInsertionsBeta, particularly those annotated as Alu, L1 and SINE-Variable number of tandem repeats-Alu (SVA), as these elements are still active in the human genome (KONKEL and BATZER 2010). Purely by genomic location, we found that 214 HuRef Standard-specific insertions overlapped a CG Alu entry, 70 with L1, and 8 with SVA (Supplementary Table 6). Next, we performed a similar analysis for calls annotated as hypervariable or invariant in the file CNV/cnvSegmentsDiploidBeta. Here, we found 24 HuRef Standard-specific gains and 31 Standard-specific losses overlapped with CG entries annotated as hypervariable, while zero gains and three losses as invariants (Supplementary Table 7). Overall, these overlap numbers were modest, and had negligible effect in improving the concordant rate between the HuRef CG and the HuRef Standard datasets.

Analysis of variation profile of the 79 samples sequenced by Complete Genomics

Upon examination of the size distribution of variants detected in our cohort of 79 CG-sequenced genomes, we noticed similar trends as observed in the HuRef CG profile (Fig. 1A and B). Importantly, there was a high consistency in the number of small variants detected by the split-read approach. Except at the maximal sizes, there was a lower degree of variability in detecting losses than gains among samples. Among the non-redundant variant set (2,874,805 gains and 2,122,600 losses) (Figure S8), there were more individual-specific gains (63.1%) than losses (54.4%) (Figure S9 A). A stronger upward trend for the gains versus losses is shown in the plot of the number of new calls obtained with each additional sample (Figure S9 B). This trend indicates that there are still many more new variants which remain to be discovered in the population, as both curves show no sign of leveling off. Overall, our data suggests that there might be more artifacts in calling gains than losses by CG, as there was a larger variability among samples along the size distribution (Fig. 1A-B) and a greater number of singletons (Figure S9).

Tables S1-S7 are available for download at http://www.g3journal.org/lookup/suppl/doi:10.1534/g3.113.008797/-/DC1.

Table S1 Summary of variation results in several personal genomes.

Table S2 Summary information of genomes sequenced in the current study.

Table S3 Gains and losses detected in the HuRef genome by different methods.

Table S4 Summary of variation results from published population studies.

Table S5 The proportion of HuRef CG variants that were also detected in our 79 Complete Genomics-sequenced samples.

Table S6 Comparison of the HuRef Standard-only variants with records annotated as mobile element insertions by Complete Genomics.

Table S7 Comparison of the HuRef Standard-only variants with records annotated as hypervariable and invariant by Complete Genomics.

Supplementary References

Abecasis, G. R., A. Auton, L. D. Brooks, M. A. DePristo, R. M. Durbin *et al.*, 2012 An integrated map of genetic variation from 1,092 human genomes. Nature 491**:** 56-65.

Abyzov, A., A. E. Urban, M. Snyder and M. Gerstein, 2011 CNVnator: an approach to discover, genotype, and characterize typical and atypical CNVs from family and population genome sequencing. Genome Res 21**:** 974-984.

Alkan, C., J. M. Kidd, T. Marques-Bonet, G. Aksay, F. Antonacci *et al.*, 2009 Personalized copy number and segmental duplication maps using next-generation sequencing. Nat Genet 41**:** 1061-1067.

Altshuler, D. M., R. A. Gibbs, L. Peltonen, E. Dermitzakis, S. F. Schaffner *et al.*, 2010 Integrating common and rare genetic variation in diverse human populations. Nature 467**:** 52-58.

Benson, G., 1999 Tandem repeats finder: a program to analyze DNA sequences. Nucleic Acids Res 27**:** 573-580.

Chiang, D. Y., G. Getz, D. B. Jaffe, M. J. O'Kelly, X. Zhao *et al.*, 2009 High-resolution mapping of copy-number alterations with massively parallel sequencing. Nat Methods 6**:** 99-103.

Conrad, D. F., D. Pinto, R. Redon, L. Feuk, O. Gokcumen *et al.*, 2010 Origins and functional impact of copy number variation in the human genome. Nature 464**:** 704-712.

Drmanac, R., A. B. Sparks, M. J. Callow, A. L. Halpern, N. L. Burns *et al.*, 2010 Human genome sequencing using unchained base reads on self-assembling DNA nanoarrays. Science 327**:** 78-81.

Durbin, R. M., G. R. Abecasis, D. L. Altshuler, A. Auton, L. D. Brooks *et al.*, 2010 A map of human genome variation from population-scale sequencing. Nature 467**:** 1061-1073.

Hormozdiari, F., C. Alkan, E. E. Eichler and S. C. Sahinalp, 2009 Combinatorial algorithms for structural variation detection in high-throughput sequenced genomes. Genome Res 19**:** 1270-1278.

Itsara, A., H. Wu, J. D. Smith, D. A. Nickerson, I. Romieu *et al.*, 2010 De novo rates and selection of large copy number variation. Genome Res 20**:** 1469-1481.

Jakobsson, M., S. W. Scholz, P. Scheet, J. R. Gibbs, J. M. VanLiere *et al.*, 2008 Genotype, haplotype and copy-number variation in worldwide human populations. Nature 451**:** 998-1003.

Ju, Y. S., D. Hong, S. Kim, S. S. Park, S. Lee *et al.*, 2010 Reference-unbiased copy number variant analysis using CGH microarrays. Nucleic Acids Res 38**:** e190.

Kidd, J. M., G. M. Cooper, W. F. Donahue, H. S. Hayden, N. Sampas *et al.*, 2008 Mapping and sequencing of structural variation from eight human genomes. Nature 453**:** 56-64.

Kidd, J. M., T. Graves, T. L. Newman, R. Fulton, H. S. Hayden *et al.*, 2010a A human genome structural variation sequencing resource reveals insights into mutational mechanisms. Cell 143**:** 837-847.

Kidd, J. M., N. Sampas, F. Antonacci, T. Graves, R. Fulton *et al.*, 2010b Characterization of missing human genome sequences and copy-number polymorphic insertions. Nat Methods 7**:** 365-371.

Konkel, M. K., and M. A. Batzer, 2010 A mobile threat to genome stability: The impact of non-LTR retrotransposons upon the human genome. Semin Cancer Biol 20**:** 211-221.

Korbel, J. O., A. E. Urban, J. P. Affourtit, B. Godwin, F. Grubert *et al.*, 2007 Paired-end mapping reveals extensive structural variation in the human genome. Science 318**:** 420-426.

Levy, S., G. Sutton, P. C. Ng, L. Feuk, A. L. Halpern *et al.*, 2007 The diploid genome sequence of an individual human. PLoS Biol 5**:** e254.

McCarroll, S. A., F. G. Kuruvilla, J. M. Korn, S. Cawley, J. Nemesh *et al.*, 2008 Integrated detection and population-genetic analysis of SNPs and copy number variation. Nat Genet 40**:** 1166-1174.

McKernan, K. J., H. E. Peckham, G. L. Costa, S. F. McLaughlin, Y. Fu *et al.*, 2009 Sequence and structural variation in a human genome uncovered by short-read, massively parallel ligation sequencing using two-base encoding. Genome Res 19**:** 1527-1541.

Mills, R. E., C. T. Luttig, C. E. Larkins, A. Beauchamp, C. Tsui *et al.*, 2006 An initial map of insertion and deletion (INDEL) variation in the human genome. Genome Res 16**:** 1182-1190.

Mills, R. E., W. S. Pittard, J. M. Mullaney, U. Farooq, T. H. Creasy *et al.*, 2011 Natural genetic variation caused by small insertions and deletions in the human genome. Genome Res 21**:** 830-839.

Pang, A. W., J. R. MacDonald, D. Pinto, J. Wei, M. A. Rafiq *et al.*, 2010 Towards a comprehensive structural variation map of an individual human genome. Genome Biol 11**:** R52.

Perry, G. H., A. Ben-Dor, A. Tsalenko, N. Sampas, L. Rodriguez-Revenga *et al.*, 2008 The fine-scale and complex architecture of human copy-number variation. Am J Hum Genet 82**:** 685-695.

Pinto, D., K. Darvishi, X. Shi, D. Rajan, D. Rigler *et al.*, 2011 Comprehensive assessment of array-based platforms and calling algorithms for detection of copy number variants. Nat Biotechnol 29**:** 512-520.

Rothberg, J. M., W. Hinz, T. M. Rearick, J. Schultz, W. Mileski *et al.*, 2011 An integrated semiconductor device enabling non-optical genome sequencing. Nature 475**:** 348-352.

Smit, A. F., 1996-2010 RepeatMasker Open-3.0, pp.

Teague, B., M. S. Waterman, S. Goldstein, K. Potamousis, S. Zhou *et al.*, 2010 High-resolution human genome structure by single-molecule analysis. Proc Natl Acad Sci U S A 107**:** 10848-10853.

Tong, P., J. G. Prendergast, A. J. Lohan, S. M. Farrington, S. Cronin *et al.*, 2010 Sequencing and analysis of an Irish human genome. Genome Biol 11**:** R91.

Wheeler, D. A., M. Srinivasan, M. Egholm, Y. Shen, L. Chen *et al.*, 2008 The complete genome of an individual by massively parallel DNA sequencing. Nature 452**:** 872-876.

Ye, K., M. H. Schulz, Q. Long, R. Apweiler and Z. Ning, 2009 Pindel: a pattern growth approach to detect break points of large deletions and medium sized insertions from paired-end short reads. Bioinformatics 25**:** 2865-2871.
