# Supplementary material for: Performance of High-Throughput Sequencing for the Discovery of Genetic Variation Across the Complete Size Spectrum
Source: G3 (Bethesda). 2013 Nov 5;4(1):63–5. doi: 10.1534/g3.113.008797 (PMC3887540; doi:10.1534/g3.113.008797)
Supplement: Supporting Information [file supp_g3.113.008797_FigureS1.pdf]

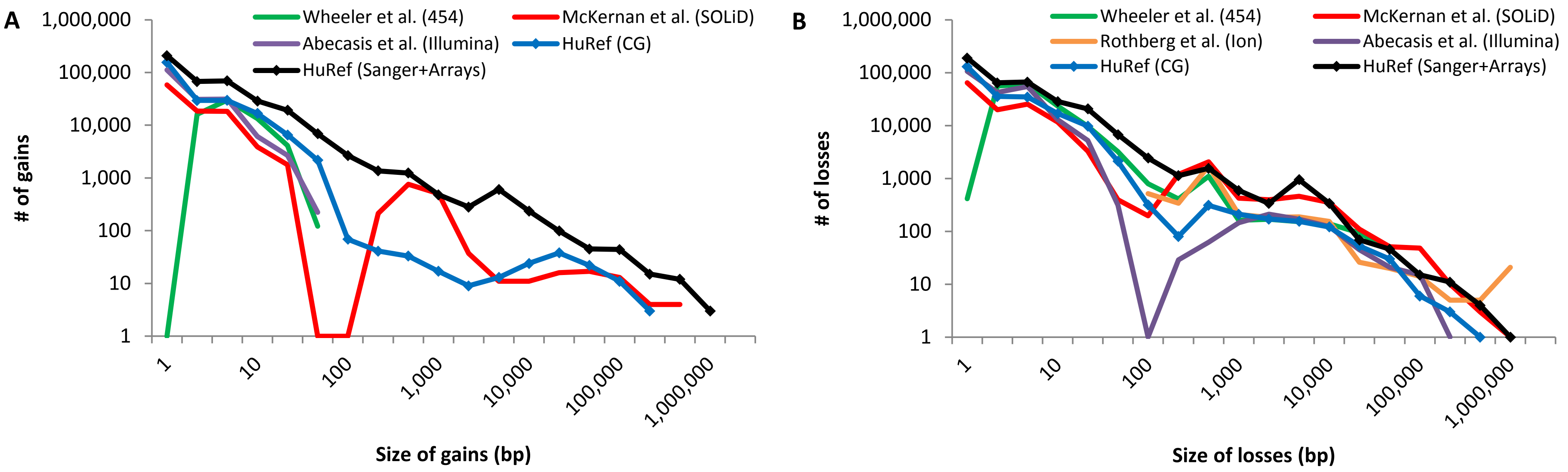

Figure S1 The size distributions of reported DNA gains and losses in published personal genome sequencing studies. These diagrams show the relative uniformity of CG variants across the size spectrum. In Wheeler et al., insertions identified by intra-read alignment would be limited by the size of the 454 sequencing reads; hence, large insertions beyond the read length were not detected (WHEELER *et al.* 2008). McKernan et al. used SOLiD sequencing and microarrays to detect variation in a Yoruba individual NA18507 (MCKERNAN *et al.* 2009). They detected small variants based on split-reads and large variants based on mate-pair mapping and microarrays, but failed to find medium size gains. Rothberg et al. performed whole genome sequencing using the Ion Torrent technology, but only reported deletions of at least 50 bp in size (ROTHBERG *et al.* 2011). Primarily sequenced by Illumina, Abecasis and colleagues detected variation in the sample NA18507 using a multitude of calling algorithms (ABECASIS *et al.* 2012). However, for large variation, only deletions were reported. The number of calls is also listed in Table S1. From these figures, CG yielded the most consistent calling pattern across the size spectrum among HTS genomes, thus justifying our selection of CG for comparison in this current study.
