# Supplementary material for: Performance of High-Throughput Sequencing for the Discovery of Genetic Variation Across the Complete Size Spectrum
Source: G3 (Bethesda). 2013 Nov 5;4(1):63–5. doi: 10.1534/g3.113.008797 (PMC3887540; doi:10.1534/g3.113.008797)
Supplement: Supporting Information [file supp_g3.113.008797_FigureS3.pdf]

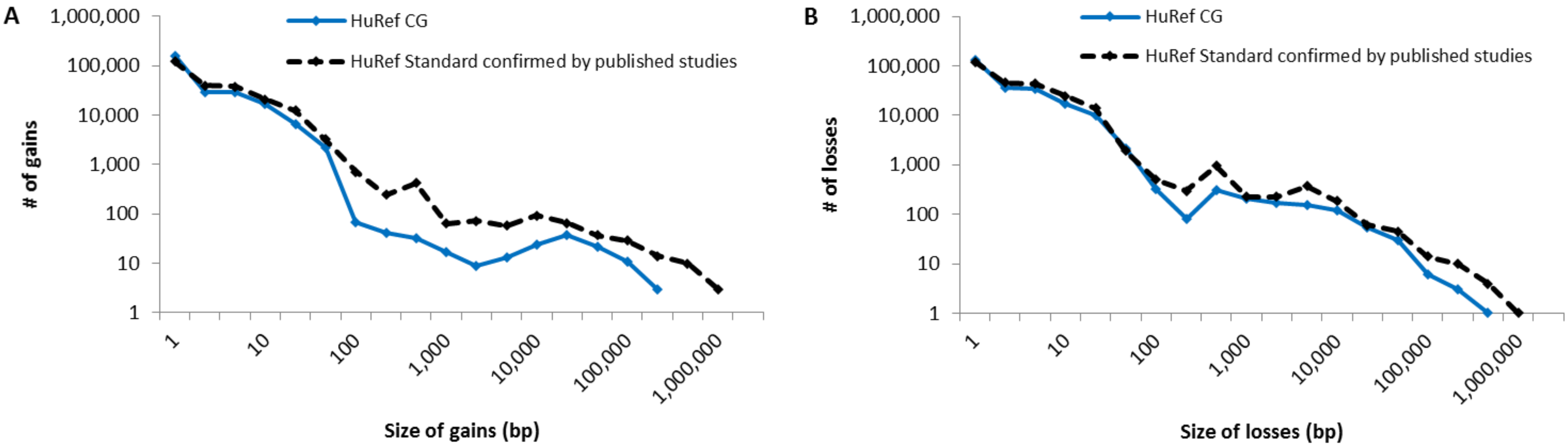

Figure S3 The size distribution of HuRef CG variation and HuRef Standard variation that was confirmed by published studies. The distributions for gains and losses are shown in plots (A) and (B), respectively. Note the resemblance of both plots to Figure 1 A and B, and that the confirmed HuRef Standard curves were consistently equal to or above the HuRef CG curves.
