# Supplementary material for: Performance of High-Throughput Sequencing for the Discovery of Genetic Variation Across the Complete Size Spectrum
Source: G3 (Bethesda). 2013 Nov 5;4(1):63–5. doi: 10.1534/g3.113.008797 (PMC3887540; doi:10.1534/g3.113.008797)
Supplement: Supporting Information [file supp_g3.113.008797_FigureS4.pdf]

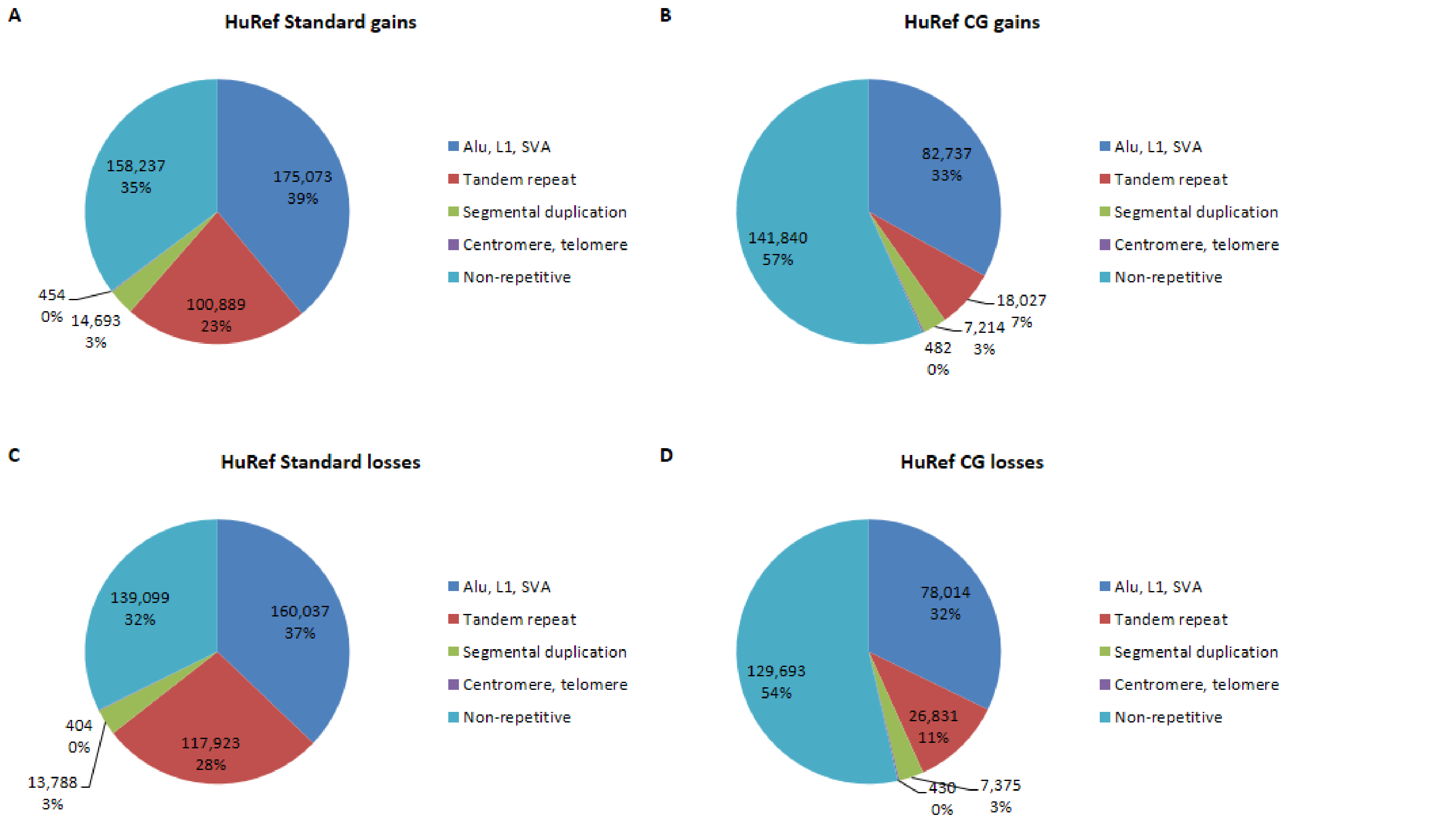

Figure S4 Proportion of HuRef Standard and HuRef CG gains and losses residing in repetitive regions. (A) The proportion of HuRef Standard gains residing in retrotransposable repeats, tandem repeats, segmental duplications, centromeric and telomeric repeats, and non-repetitive regions. (B) HuRef CG gains and proportion of repeats. (C) HuRef Standard losses. (D) HuRef CG losses. Note the under-representation of HuRef CG calls in retrotransposons, tandem repeats and segmental duplications when compared to the HuRef Standard profile. Finally, the sum of the numbers reported in these plots exceeds the total number of variants, because it is possible that one variant overlap with more than two types of repeats.
