# Supplementary material for: Performance of High-Throughput Sequencing for the Discovery of Genetic Variation Across the Complete Size Spectrum
Source: G3 (Bethesda). 2013 Nov 5;4(1):63–5. doi: 10.1534/g3.113.008797 (PMC3887540; doi:10.1534/g3.113.008797)
Supplement: Supporting Information [file supp_g3.113.008797_FigureS5.pdf]

HuRef CG total	
# gains	241,033
# losses	230,737

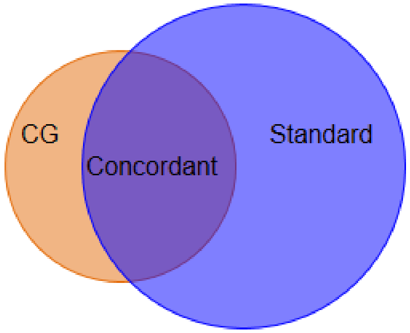

HuRef Standard total	
# gains	408,403
# losses	383,470

HuRef CG-specific*	
# gains (%)	98,665 (40.93)
# losses (%)	70,345 (30.49)

Concordant*	
# gains (%)	142,368 (59.07)
# losses (%)	160,392 (69.51)

HuRef Standard-specific**	
# gains (%)	265,858 (65.10)
# losses (%)	222,549 (58.04)

* Percentage is with respect to HuRef CG total
** Percentage is with respect to HuRef Standard total

Figure S5 Overall concordance statistics between HuRef Standard and HuRef CG variation sets.
