# Supplementary material for: Performance of High-Throughput Sequencing for the Discovery of Genetic Variation Across the Complete Size Spectrum
Source: G3 (Bethesda). 2013 Nov 5;4(1):63–5. doi: 10.1534/g3.113.008797 (PMC3887540; doi:10.1534/g3.113.008797)
Supplement: Supporting Information [file supp_g3.113.008797_FigureS6.pdf]

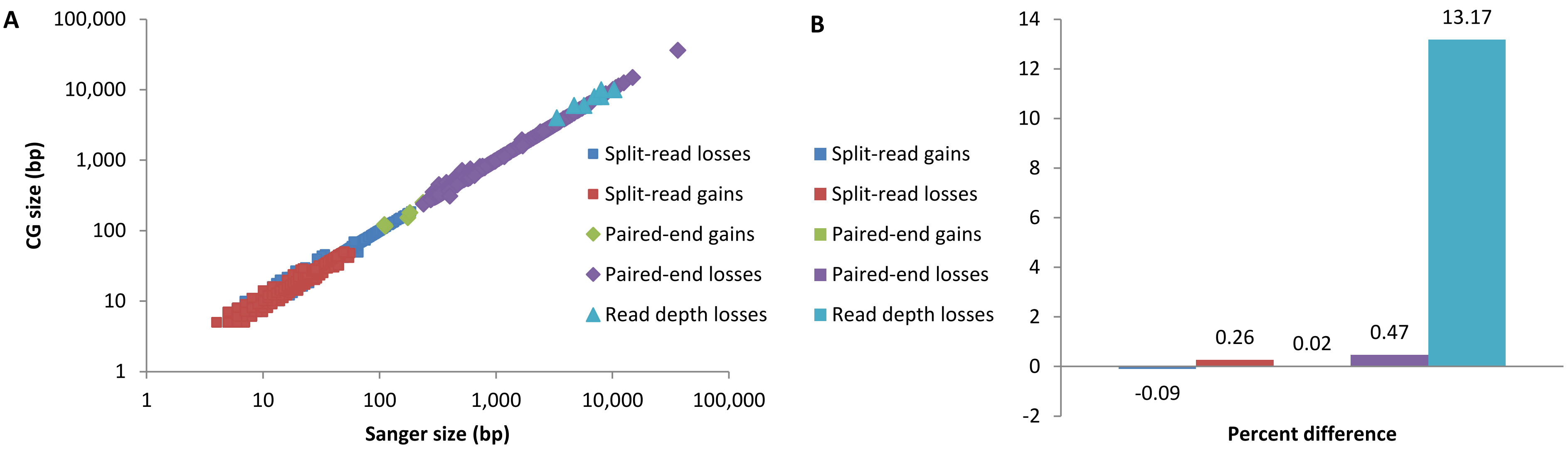

Figure S6 Complete Genomics variant breakpoint estimation. (A) shows the tight size correlation between HuRef CG variants (> 5bp) and the corresponding breakpoint-refined HuRef Standard variants, and (B) displays the average percentage of size difference between HuRef CG and HuRef Standard.
