# Supplementary material for: Performance of High-Throughput Sequencing for the Discovery of Genetic Variation Across the Complete Size Spectrum
Source: G3 (Bethesda). 2013 Nov 5;4(1):63–5. doi: 10.1534/g3.113.008797 (PMC3887540; doi:10.1534/g3.113.008797)
Supplement: Supporting Information [file supp_g3.113.008797_FigureS7.pdf]

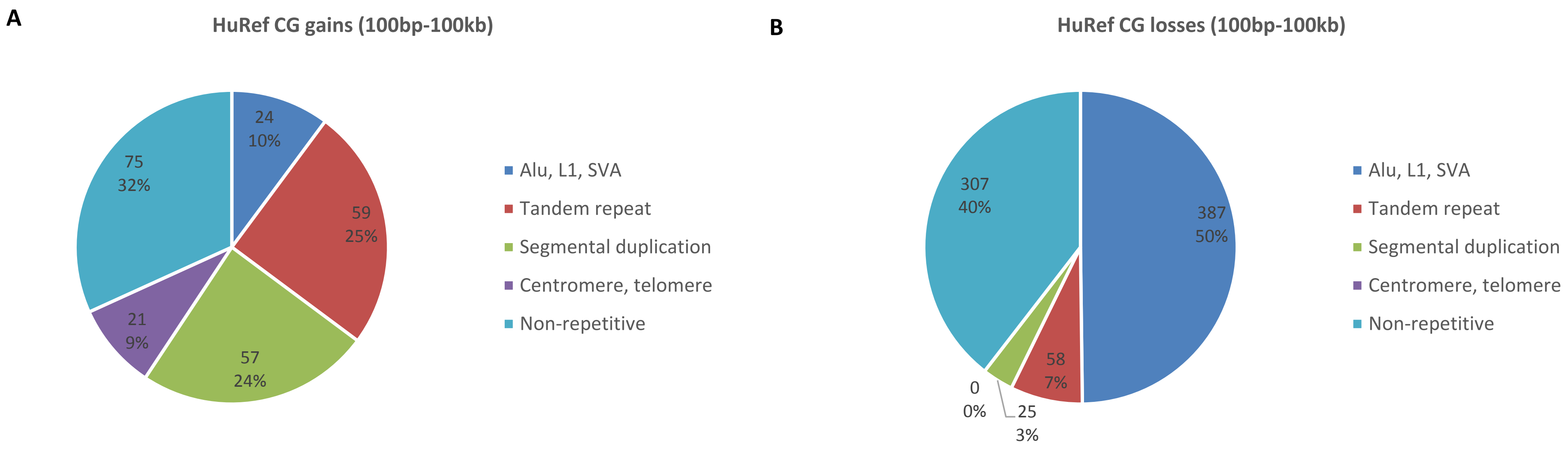

Figure S7 The percentage of HuRef CG gains and losses between 100 bp and 100 kb residing in retrotransposable repeats, tandem repeats, segmental duplications, centromeric and telomeric repeats. The depletion of insertions reported in retrotransposable elements has been known and this situation has also been acknowledged by Complete Genomics. Surprisingly, there were significant elevations in the number of HuRef CG gains versus losses residing in other types of repeats (tandem repeat and segmental duplication). However, these repeats could also be problematic for short-read alignments. The enrichment of gains over losses found in these loci could explain why a lower CG paired-end and CG read depth confirmation rate was observed for gains compared to losses. (A) HuRef CG gains. (B) HuRef CG losses.
