# Supplementary material for: Performance of High-Throughput Sequencing for the Discovery of Genetic Variation Across the Complete Size Spectrum
Source: G3 (Bethesda). 2013 Nov 5;4(1):63–5. doi: 10.1534/g3.113.008797 (PMC3887540; doi:10.1534/g3.113.008797)
Supplement: Supporting Information [file supp_g3.113.008797_FigureS8.pdf]

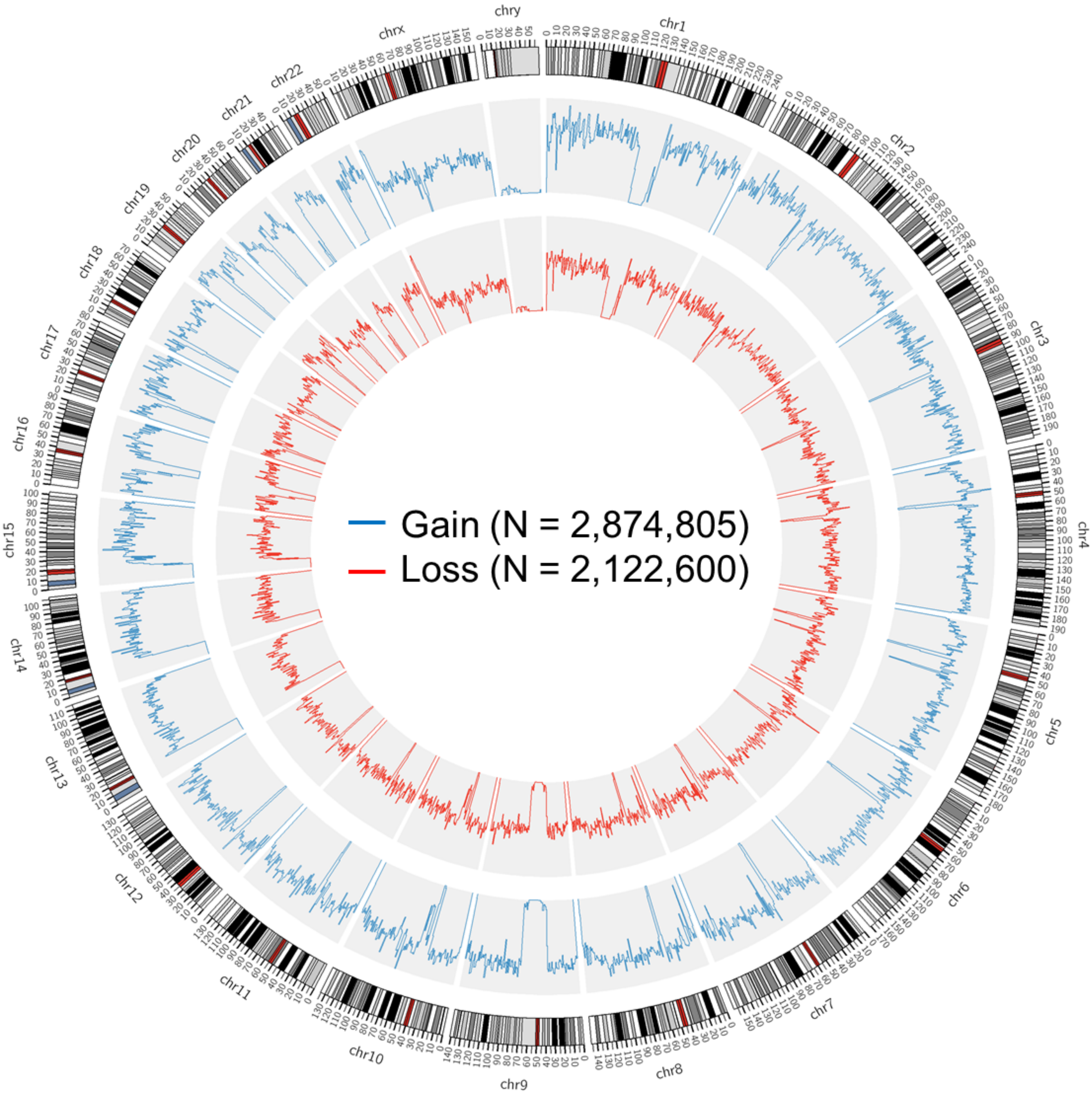

Figure S8 Density distribution of non-redundant Complete Genomics variants found in our 79-sample cohort along chromosomal locations. The density of gains is presented by the blue track, while losses by the red (number of non-redundant variant per megabase). This provides a snapshot of the distribution of calls along chromosomes. For example, the overall profiles between gain and loss are similar, and the density at repetitive loci (centromeres, telomeres, p-arm of acrocentric chromosomes, chromosome Y) and are notably low. Centromeres are shown in red in the ideogram.
