# Supplementary material for: Performance of High-Throughput Sequencing for the Discovery of Genetic Variation Across the Complete Size Spectrum
Source: G3 (Bethesda). 2013 Nov 5;4(1):63–5. doi: 10.1534/g3.113.008797 (PMC3887540; doi:10.1534/g3.113.008797)
Supplement: Supporting Information [file supp_g3.113.008797_FigureS9.pdf]

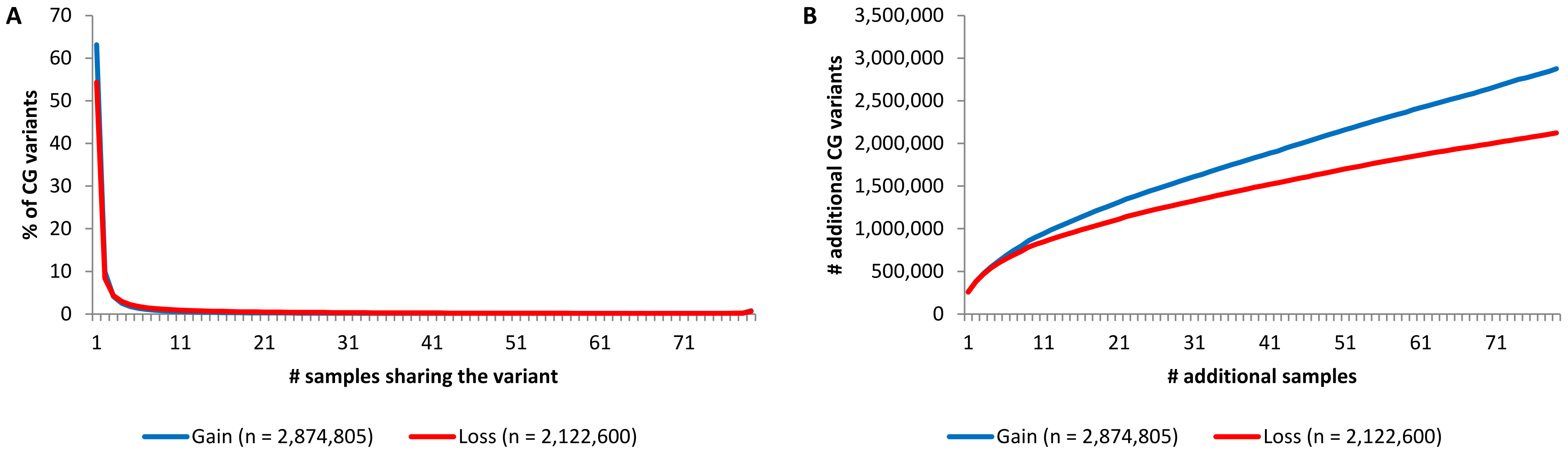

Figure S9 Frequency of gains and losses detected in the 79 Complete Genomics cohort. (A) The frequency of gains and losses found in the 79 samples sequenced by Complete Genomics. (B) Cumulative frequency distribution showing the number new variants obtained with the sequencing of each additional DNA sample.
