# Supplementary material for: Performance of High-Throughput Sequencing for the Discovery of Genetic Variation Across the Complete Size Spectrum
Source: G3 (Bethesda). 2013 Nov 5;4(1):63–5. doi: 10.1534/g3.113.008797 (PMC3887540; doi:10.1534/g3.113.008797)
Supplement: Supporting Information [file supp_g3.113.008797_FigureS10.pdf]

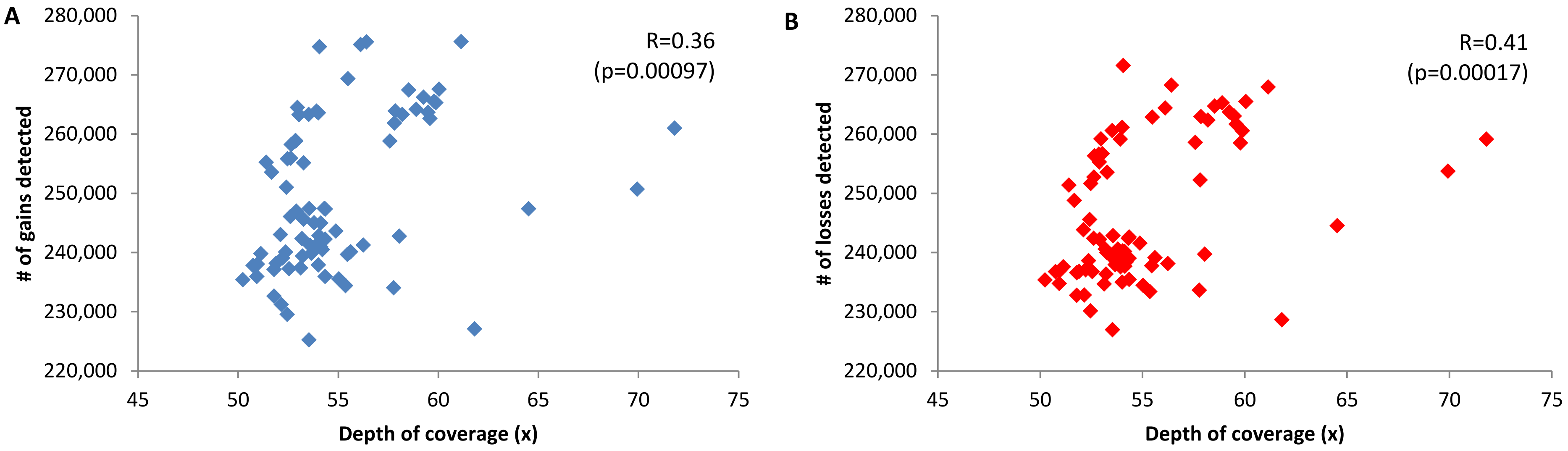

Figure S10 Positive correlation between the depth of coverage and the number of gains and losses detected among the 80 samples sequenced by CG. (A) Gains. (B) Losses.
