# Supplementary material for: Performance of High-Throughput Sequencing for the Discovery of Genetic Variation Across the Complete Size Spectrum
Source: G3 (Bethesda). 2013 Nov 5;4(1):63–5. doi: 10.1534/g3.113.008797 (PMC3887540; doi:10.1534/g3.113.008797)
Supplement: Supporting Information [file supp_g3.113.008797_FileS1.pdf]

**File S1**

**Supplementary Materials and Methods**

**Complete Genomics sequencing experiment and data analysis**

As required by Complete Genomics (CG) (Drmanac *et al.* 2010), 10 ug of non-degraded DNA was provided for sequencing. The sequencing experiments and variation-detection of the genomes of Craig Venter (HuRef) (Levy *et al.* 2007) and 79 clinically unaffected Caucasians were performed in house at CG. Paired-end reads were aligned to the Genome Reference Consortium human genome reference GRCh37. A summary of the sequencing experiments is shown in Supplementary Table 2.

The variant calls were extracted from CG files using custom Perl scripts. The variation set used for this study was obtained from three primary sources: (i) The CG split-read insertion and deletion records were extracted from the masterVarBeta file. (ii) The paired-end set was obtained from the SV/highConfidenceSVEventsBeta file, and only deletion, distal and tandem duplications were extracted. (iii) The read depth data was taken from the CNV/cnvSegmentsDiploidBeta file. The records of hypervariable or invariant were not included in our final data set for subsequent analysis, because the assignment of calls as gain or loss was not provided. However, we did perform additional analysis incorporating these calls in the HuRef CG (the HuRef CG) dataset to try to improve the concordant rate with the HuRef Sanger + Array standard (the HuRef Standard) (Levy *et al.* 2007; Pang *et al.* 2010). See Supplementary Results *Analysis of MEI, hypervariable and invariant CG records* section for more information.

CG also explicitly searched for potential mobile element insertions (MEIs), and they were listed in MEI/mobileElementInsertionsBeta file. We did not include these calls in the final variant set, as the variant size was not annotated. Although the file did annotate the start and end of insertion fragment within the consensus sequence of the mobile element, the information might not necessarily represent the size of the complete insertion sequence. There could be sequences within an insertion fragment that could not be aligned to the mobile element consensus sequence. So, entries in the file were not included in the CG final variant set. Nonetheless, we did compare a subset of these MEI calls in the HuRef sample with the calls in the HuRef Standard, and the results are listed in Supplementary Results *Analysis of MEI, hypervariable and invariant CG records* section.

**Non-redundant Complete Genomics variant set generation**

To generate a non-redundant set of variation, we combined the split-read, paired-end and read depth records. First we searched for overlap between the paired-end and read depth sets requiring that the variants to be the same type (i.e. duplication or deletion) and that they shared a minimum of 50 % reciprocal size overlap. Next, we used the same criteria to merge this dataset with the split-read calls. For those calls that were determined to be the same variant, we recorded the one with a better size/boundary estimate, with preference given to split-read, then paired-end, then read depth.

**Population reference data set**

We compiled a non-redundant set of calls from 18 published studies (Jakobsson *et al.* 2008; Kidd *et al.* 2008; McCarroll *et al.* 2008; Perry *et al.* 2008; Wheeler *et al.* 2008; Alkan *et al.* 2009; Altshuler *et al.* 2010; Conrad *et al.* 2010; Durbin *et al.* 2010; Itsara *et al.* 2010; Ju *et al.* 2010; Kidd *et al.* 2010a; Kidd *et al.* 2010b; Teague *et al.* 2010; Tong *et al.* 2010; Mills *et al.* 2011; Pinto *et al.* 2011; Abecasis *et al.* 2012) for comparison with the HuRef data. A summary of these studies can be found in Supplementary Table 4. This list consisted of studies that used a variety of variation-detection methodologies, ranging from NGS sequencing, Sanger sequencing and Sanger fosmid mate-pair mapping, high density SNP genotyping microarrays, high resolution comparative genome hybridization microarrays, and optical mapping. To determine if two calls correspond to the same underlying event, we used a strict 70 % reciprocal size overlap criterion.

**Genomic features data set**

The positions of retrotransposable, centromeric and telomeric repeats were taken from Repeat Masker (Smit 1996-2010). Segmental duplications information was obtained from the University of California, Santa Cruz (UCSC) database. Tandem repeats annotation was taken from Tandem Repeats Finder (Benson 1999).
