# Supplementary material for: Performance of High-Throughput Sequencing for the Discovery of Genetic Variation Across the Complete Size Spectrum
Source: G3 (Bethesda). 2013 Nov 5;4(1):63–5. doi: 10.1534/g3.113.008797 (PMC3887540; doi:10.1534/g3.113.008797)
Supplement: Supporting Information [file supp_g3.113.008797_FileS2.pdf]

File S2

Supplementary Results

Examination of HuRef variants called by different Complete Genomics detection approaches

We investigated the confirmation rate among the detection approaches of CG. It used three primary approaches to detect non-SNP variants: paired-end, split-read and read depth. The first approach used by CG is the paired-end mapping method. When paired sequences from an insert library of defined sizes created from genomic DNA from a test individual are sequenced, they are then aligned to a reference assembly. The distance between the pair is then compared with the expected size of the insert. Any discrepancy in distance between the observed and expected size of the insert would indicate a putative insertion or deletion (KORBEL *et al.* 2007; KIDD *et al.* 2008; HORMOZDIARI *et al.* 2009). The split-read approach will detect insertions and deletions by identifying intra-alignment gaps (MILLS *et al.* 2006; YE *et al.* 2009). Finally, the read depth approach is used to identify duplications where there is a significantly elevated number of alignments compared to diploid regions, while deleted regions are identified when there is a significantly reduced number of mapped reads (CHIANG *et al.* 2009; ABYZOV *et al.* 2011).

Among the three detection approaches employed by CG, the split-read approach was used to call small variants, paired-end approach for medium to large variants, and read depth approach for large ones (Fig. 2, Supplementary Table 3). In the HuRef CG dataset, there was no overlap between variants called by the split-read and the other approaches, mainly because the size of split-read calls was below others' detection limits. As for the larger variation, the concordant rate between paired-end and read depth sets was modest; their consensus was two for the gains and 45 for the losses. Interestingly, there was an elevated level of overlap with segmental duplications and tandem repeats in the gains compared to the losses in the 100 bp to 100 kb size range (Figure S7). The difficulty to localize variants by short reads mapping to repeats could explain the lower cross-detection-approach confirmation rate of the gains.

Estimation of Complete Genomics variant-detection sensitivity

We compared the HuRef Standard and HuRef CG variants with published variation studies, which constituted our population reference. These studies have used multiple variant-detection methods: high-throughput sequencing (HTS), Sanger read-trace, Sanger fosmid-end mapping, microarrays and optical mapping (Supplementary Table 4), and from these, we compiled 1,637,756 non-redundant gains and 2,113,933 non-redundant losses, whose size distributions are displayed in Supplementary Figure 2. The uneven size distributions of the population reference suggested a detection bias to variants of certain sizes. Even though we incorporated studies using a wide range of genomic approaches, there were still shortcomings at certain size ranges, especially between 100 bp to 10 kb. Despite the notable shortcomings in the population reference, we compared it with the HuRef Standard and HuRef CG variation profiles.

First, the size distribution curves representing those HuRef Standard variants also detected in the population reference were consistently at or above the overall HuRef CG curves, across the entire size spectrum (Supplementary Fig. 3), indicating that there were variants missing in the HuRef CG profile.

Second, we examined the proportion of HuRef CG-only and HuRef Standard-only variants confirmed by the population reference. We found that a similar proportion of HuRef CG-only (44.9 % gains and 61.3 % losses) and HuRef Standard-only variants (40.6 % gains and 50.0 % losses) were in accord with the population reference. So, both data sets contained genuine variants that were undetectable by the other. We want to emphasize that our usage of population reference was for estimating sensitivity, not false discovery. Any discordance could be due to sample difference or methodological difference in variation detection. Furthermore, the population reference was incomplete, with a notable plateau at the 100 bp to 10 kb range (Supplementary Fig. 2), and this in turn could lower the concordant rate in both HuRef variants sets.

Estimation of Complete Genomics variant-detection specificity and false discovery rate

While there was a good overall concordance (64.2%) for the HuRef CG calls with the HuRef Standard, the specificity to detect gains was lower than that for losses. We found that 59.1% (142,368/241,033) of gains and 69.5% (160,392/230,737) of losses called by HuRef CG were concordant (70% reciprocal size overlap) with the HuRef Standard (Supplementary Fig. 5).

Since there could be variants discoverable only by the CG technology, we refined the false discovery rate by further comparing the HuRef CG profile with those from the other 79 CG-sequenced genomes in this study. By comparing with samples that were also sequenced on the same platform, we removed the confounding issue of methodological difference in variation detection. Among the HuRef CG-only variants not detectable by HuRef Standard, there were *(I)* 28,020 gains and 9,904 losses that were also not seen in the other 79 samples (Supplementary Table 5). These calls could be false discoveries in the HuRef CG experiment or rare/private HuRef CG variants undetectable by the HuRef Standard.

On the other hand, among the set of HuRef CG variants that were concordant with the HuRef Standard, only *(II)* 937 (of 142,368; or 0.66%) gains and 1,996 (of 160,392; or 1.24%) losses were also not supported by the 79 individuals (Supplementary Table 5), and we reasoned that these proportions of calls should represent high confidence rare/private HuRef variants.

We then applied percentage from *(II)* to the calls in HuRef CG-only (not confirmed in HuRef Standard) and estimated the expected number of these calls to be private.

98,665 gains * 0.66% = 651 expected number of rare/private gains

70,345 losses * 1.24% = 872 expected number of rare/private losses*…(III)*

Therefore by taking the difference between *(I)* and *(III)*, we estimated that 27,369 or 11.35% (= [28,020 – 651] / 241,033 * 100) HuRef CG gains, and 9,032 or 3.91% (= [9,904 – 872] / 230,737 * 100) HuRef CG losses could be false discoveries. Once again, similar to Supplementary Figure 5, we observed that the specificity to detect gains was lower than losses.

However, we emphasize that these are only estimates, as there are other types of false calls unaccounted for. One type is false variation repeatedly called in all CG experiments. One such example was the 16.8 Mb HuRef deletion artifact found by paired-end mapping method of CG, which was also repeatedly called in all 79 genomes in our sample cohort (Supplementary Table 3). This large-size call was likely false as it had not been confirmed by the HuRef Standard or cytogenetic experiments (LEVY *et al.* 2007).

Finally, we examined additional HuRef CG records for concordance (mobile element insertions, hypervariable and invariant calls), but the improvement was insignificant. See the section *Analysis of MEI, hypervariable and invariant Complete Genomics records* in Supplementary Results.

Analysis of MEI, hypervariable and invariant Complete Genomics records

From our concordance analysis of comparing the HuRef CG data with the HuRef Standard data, we determined that 142,368 (59.1 %) gains and 160,392 (69.5 %) losses called by CG were also concordant with the HuRef Standard. In other words, there were 265,858 gains and 222,549 losses specific to the HuRef Standard and undetectable by CG. We then included additional potentially relevant but lower confidence CG calls. For the following exercises, we examined the number CG calls that might correspond to calls that were HuRef Standard-only.

First, we examined the CG MEI variants in the file MEI/mobileElementInsertionsBeta, particularly those annotated as Alu, L1 and SINE-Variable number of tandem repeats-Alu (SVA), as these elements are still active in the human genome (KONKEL and BATZER 2010). Purely by genomic location, we found that 214 HuRef Standard-specific insertions overlapped a CG Alu entry, 70 with L1, and 8 with SVA (Supplementary Table 6). Next, we performed a similar analysis for calls annotated as hypervariable or invariant in the file CNV/cnvSegmentsDiploidBeta. Here, we found 24 HuRef Standard-specific gains and 31 Standard-specific losses overlapped with CG entries annotated as hypervariable, while zero gains and three losses as invariants (Supplementary Table 7). Overall, these overlap numbers were modest, and had negligible effect in improving the concordant rate between the HuRef CG and the HuRef Standard datasets.

Analysis of variation profile of the 79 samples sequenced by Complete Genomics

Upon examination of the size distribution of variants detected in our cohort of 79 CG-sequenced genomes, we noticed similar trends as observed in the HuRef CG profile (Fig. 1A and B). Importantly, there was a high consistency in the number of small variants detected by the split-read approach. Except at the maximal sizes, there was a lower degree of variability in detecting losses than gains among samples. Among the non-redundant variant set (2,874,805 gains and 2,122,600 losses) (Figure S8), there were more individual-specific gains (63.1%) than losses (54.4%) (Figure S9 A). A stronger upward trend for the gains versus losses is shown in the plot of the number of new calls obtained with each additional sample (Figure S9 B). This trend indicates that there are still many more new variants which remain to be discovered in the population, as both curves show no sign of leveling off. Overall, our data suggests that there might be more artifacts in calling gains than losses by CG, as there was a larger variability among samples along the size distribution (Fig. 1A-B) and a greater number of singletons (Figure S9).
